# Supplementary material for: Factors associated with continuation of hormonal contraceptives among married women of reproductive age in Gilgit, Pakistan: a community-based case–control study
Source: BMJ Open. 2023 Nov 23;13(11):e075490. doi: 10.1136/bmjopen-2023-075490 (PMC10668236; doi:10.1136/bmjopen-2023-075490)
Supplement: Author's manuscript [file bmjopen-2023-075490.draft_revisions.pdf]

BMJ Open

BMJ Open is committed to open peer review. As part of this commitment we make the peer review history of every article we publish publicly available.

When an article is published we post the peer reviewers' comments and the authors' responses online. We also post the versions of the paper that were used during peer review. These are the versions that the peer review comments apply to.

The versions of the paper that follow are the versions that were submitted during the peer review process. They are not the versions of record or the final published versions. They should not be cited or distributed as the published version of this manuscript.

BMJ Open is an open access journal and the full, final, typeset and author-corrected version of record of the manuscript is available on our site with no access controls, subscription charges or pay-per-view fees (http://bmjopen.bmj.com).

If you have any questions on BMJ Open's open peer review process please email info.bmjopen@bmj.com

BMJ Open

Factors associated with continuation of hormonal contraceptives among married women of reproductive age in Gilgit- Pakistan: a community-based case control study

Journal:	*BMJ Open*
Manuscript ID	bmjopen-2023-075490
Article Type:	Original research
Date Submitted by the Author:	10-May-2023
Complete List of Authors:	sahibjan, Fazila; The Aga Khan University, Center of excellence for trauma and emergencies<br>Saleem, Sarah; Aga Khan University, Community Health Sciences<br>Rozi, Shafquat; Aga Khan University, Community Health Sciences<br>Tikmani, Shiyam; Aga Khan University, Community Health Sciences
Keywords:	SEXUAL MEDICINE, REPRODUCTIVE MEDICINE, GYNAECOLOGY, Subfertility < GYNAECOLOGY, Health Services Accessibility

SCHOLARONE™
Manuscripts

I, the Submitting Author has the right to grant and does grant on behalf of all authors of the Work (as defined in the below author licence), an exclusive licence and/or a non-exclusive licence for contributions from authors who are: i) UK Crown employees; ii) where BMJ has agreed a CC-BY licence shall apply, and/or iii) in accordance with the terms applicable for US Federal Government officers or employees acting as part of their official duties; on a worldwide, perpetual, irrevocable, royalty-free basis to BMJ Publishing Group Ltd ("BMJ") its licensees and where the relevant Journal is co-owned by BMJ to the co-owners of the Journal, to publish the Work in this journal and any other BMJ products and to exploit all rights, as set out in our licence.

The Submitting Author accepts and understands that any supply made under these terms is made by BMJ to the Submitting Author unless you are acting as an employee on behalf of your employer or a postgraduate student of an affiliated institution which is paying any applicable article publishing charge ("APC") for Open Access articles. Where the Submitting Author wishes to make the Work available on an Open Access basis (and intends to pay the relevant APC), the terms of reuse of such Open Access shall be governed by a Creative Commons licence – details of these licences and which Creative Commons licence will apply to this Work are set out in our licence referred to above.

Other than as permitted in any relevant BMJ Author's Self Archiving Policies, I confirm this Work has not been accepted for publication elsewhere, is not being considered for publication elsewhere and does not duplicate material already published. I confirm all authors consent to publication of this Work and authorise the granting of this licence.

For peer review only

Factors associated with continuation of hormonal contraceptives among married women of reproductive age in Gilgit- Pakistan: a community-based case control study

Fazila Bibi[1*], Sarah Saleem[2], Shafquat Rozi[3], Shiyam Sunder[4]

[1]Center of Excellence for Trauma and Emergencies, Aga Khan University, Karachi, Pakistan

[2]Community Health Sciences, Aga Khan University, Karachi, Pakistan

[3]Community Health Sciences, Aga Khan University, Karachi, Pakistan

[4]Community Health Sciences, Aga Khan University, Karachi, Pakistan

Author information:

Fazila Bibi (FB)- Fazila.sahibjan@aku.edu

Sarah Saleem (SS)- sarah.saleem@aku.edu

Shafquat Rozi (SR)- Shafquat.rozi@aku.edu

Shiyam Sunder (SS) Shiyam.sunder@aku.edu

Correspondence to Natasha Shaukat: fazila.sahibjan@aku.edu

Centre of Excellence for Trauma and Emergencies, Aga Khan University, Karachi,

Address: Stadium Road, P.O. Box-3500, Karachi (74800), Pakistan.

Tel: 92-21-3486-4837

Fax: 92-21-34932095

ABSTRACT

For peer review only - http://bmjopen.bmj.com/site/about/guidelines.xhtml

Background

Worldwide continuation of contraceptive use is affected by service delivery, biological and sociodemographic factors. This study aimed to determine the factors associated with continuation of hormonal contraceptive methods among married women of Gilgit, Pakistan at least six months after their initiation.

Methods

An unmatched case control study was conducted in the community settings of district Gilgit, Pakistan from April 1, 2021, to July 30, 2021. The cases were married women of reproductive age who at the time of interview were using a hormonal method of contraception for at least last six months continuously, and controls were married women of reproductive age who had used hormonal method in the past and currently were using a non-hormonal method for at least last six months. Simple and multiple logistic regression method was used to identify factors associated with continuation of hormonal methods of contraception.

Results

The factors significantly associated with continuous use of hormonal contraceptive methods for our sample from Gilgit were family planning center at the walking distance from home (AOR 6.33, 95% CI, 3.74-10.71), satisfaction with current method in use (AOR 3.64 95% CI, 2.06-6.44), visits to the family planning center to avail services (AOR 1.86 95% CI, 1.07-3.45) and relatively older age of women (AOR 1.07, 95% C1, 1.02-1.12). In addition, women with formal education (AOR 0.27 95% CI, 0.12-0.6), were less likely to use a modern contraceptive method.

Conclusion

Continuation of a hormonal method use was associated with easy access to family planning centers, satisfaction with the current method use and frequent visits to the family planning centers. Continuation of hormonal method use was also seen in women with low education status. The importance of the presence of family planning centers in close proximity to residential areas cannot be emphasized more. This not only provides easy access to FP

For peer review only - http://bmjopen.bmj.com/site/about/guidelines.xhtml

methods, but also reassures women for continuation of modern methods when they face any unpleasant effects while using these.

Key words: Hormonal Contraceptives, continuation, women of reproductive age

For peer review only

Strengths and Limitations

For peer review only - http://bmjopen.bmj.com/site/about/guidelines.xhtml

- Recruitment of the study participants was based on identification from lady health workers records as they do frequent visits and keep their records updated and we reconfirmed from the women before recruiting.
- This was a robust method leading to decrease chance of misclassification of the outcome status of the participants.
- Case to control ratio was kept 1:2 which is more likely to represent experience of general population and similar data collection tool was used to collect data from cases and controls.
- Major limitation of the study was the subjective assessment of walking distance due to unavailability of internet access in all union councils of district Gilgit. However, those women who never used transport to reach FP is supportive of giving an affirmation of the proximity of FP centers from their homes.
- This study is generalizable to areas that are geographically like district Gilgit.

For peer review only

Introduction

For peer review only - http://bmjopen.bmj.com/site/about/guidelines.xhtml

Access to safe and effective contraceptives is the right of every woman and man irrespective of their religion, or socio-economic status, region, or country, so that, they can make an informed choice of their preferred type of method they would like to use (1). The trends of contraceptive use, its discontinuation and switching a method are significant identifiers of how well family planning programs are fulfilling the needs of family planning of reproductive age women and couples in a country (2). Worldwide 922 million women of reproductive age use any form of contraceptives (3), however, there are still 12 % of the women who want to avoid pregnancy and are not using any method of contraception (4). High fertility rates and low contraceptive prevalence (CPR) rates are the main elements of exponential population growth in South Asian countries (5). In Pakistan, the high fertility rate and low use of contraceptives are contributing factors to poor reproductive health indicators for maternal mortality. Furthermore, discontinuation of contraception has been found to be one of the major reasons of induced abortions in Pakistan. The difference in ever use of modern contraceptive and current use indicates that women who had started using modern contraceptives were giving them up and not returning (4). Multi-indicator cluster survey of Pakistan (MICS 2016-17) reported that in Gilgit Baltistan (GB) 38.1% of married women of reproductive age had access to contraception, of these 32.1% use one of the modern contraceptive methods (6).

Contraceptive continuation is the process of uninterrupted usage of contraceptive methods for at least six months. Factors associated with contraceptive continuation are both methods related such as efficacy of the method, frequency of doses, and client- related such as age, education level, parity etc. (7,8). Distance of the family planning center from home and availability of different methods at the center are additional important factors contributing to continuity in use of a method. Presence of healthcare staff at the center, level of education,

For peer review only - http://bmjopen.bmj.com/site/about/guidelines.xhtml

age, number of living children and area of residence were all found to be associated with contraceptive continuation (9,10,11,12).

Information regarding predictors of continuation of hormonal contraceptives is scarce from Pakistan and information related to continuation of any contraceptive method is mostly unavailable in Pakistan. Gilgit Baltistan is situated in Northern part of Pakistan which covers an area of over 72,971 KM^2 and is highly hilly and mountainous. Gilgit is the capital and largest district with estimated population of 216,760 (13). Seventy-nine percent of family planning centers with trained staff in Gilgit are in the main town while remaining are in other union councils with 4-29 % trained staff (13) as shown in figure1.

Figure1: Map of Gilgit Baltistan showing distance in Kilometers from union council to family planning centers and distribution of LHWs in public and private family planning centers.

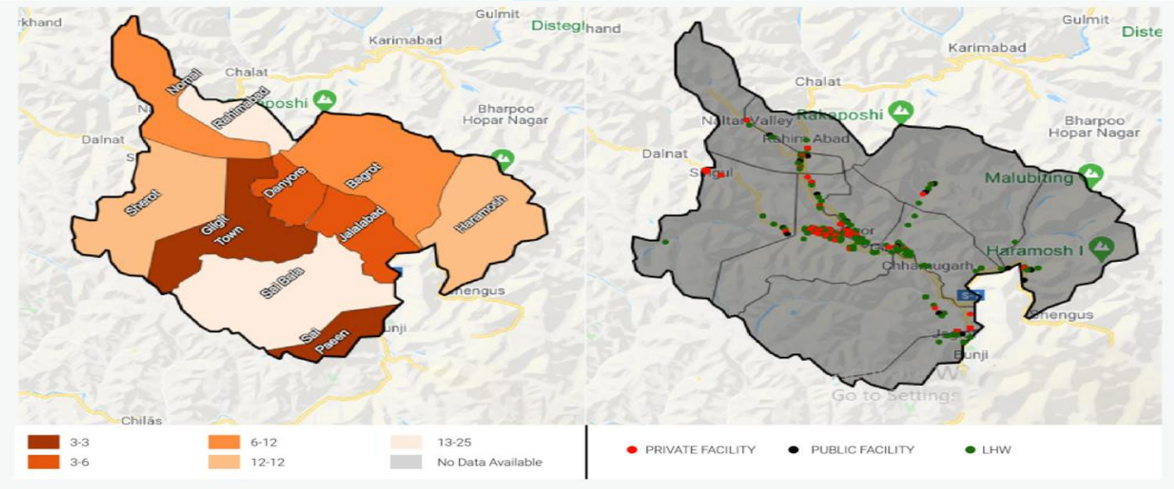

This suggests that all specialized facilities are centered in Gilgit town and women from other union councils have to travel to get specialized services and the average distance from Gilgit town to the nearest health facility is 2.7 kilometers which is far less than other union councils that are 18-24 kilometers away from the nearest health facility (13). Considering the high efficacy of the hormonal methods to prevent pregnancy, we were interested in identifying region-specific (Gilgit) factors that were associated with the continuation of hormonal contraceptives.

For peer review only - http://bmjopen.bmj.com/site/about/guidelines.xhtml

Methods

We use a community-based unmatched case control study design, with ratio of cases to control as 1:2. Cases were married women of reproductive age who were currently using a hormonal method of contraception for at least last six months, and controls were married women of reproductive age who had ever used a hormonal method but now are using a non-hormonal method at least for last six months. This study was carried out in the community setting of Gilgit, Pakistan. Gilgit is the largest district of the Baltistan territory and consists of ten union councils and constitutes 19 % of total population of Baltistan. We collected data from the catchment population of LHWs of 3 union councils Gilgit town, Danyore, and Nomal. Hormonal method users were sexually active women who were using one of the hormonal contraceptive methods i.e., pills, injectables or implants for at least last six months without disruption at the time of enrolment in the study and Controls were sexually active married women of reproductive age, with history of using a hormonal method but stopped it before six months after initiating it and switched to a non-hormonal contraceptive method for at least last six months. We excluded women using intrauterine contraceptive device (IUCD), as there are of two types (hormonal and non-hormonal) methods and women might not know the type of IUCD she was using. Women who were using permanent method of contraception were also excluded. The women were identified through the records of lady health workers and the status as case or control was verified at the time of visiting home for interviews by our research assistants who were trained for the purpose. These lady workers are government employed workers and provide doorstep promotional, motivational and some basic health care services to women and children, such as iron and folic acid tablets to pregnant women and provision of condoms and pills in addition to counseling for childhood immunization, weaning diet for infants and receiving ANC (antenatal care) and skilled birth attendance. Each LHW is assigned 125 to 200 houses in different union councils of district Gilgit, they

For peer review only - http://bmjopen.bmj.com/site/about/guidelines.xhtml

do door to door visits every three months. All the households of our study participants were visited by lady health worker in last six months.

Our outcome variable was defined as ‘continuous use of hormonal contraceptive method for at least last six months and is described as a qualitative nominal variable with binary outcome as “yes” and “no”

Walking distance from home to nearest family planning center was measured subjectively by asking women if they could access nearest center on foot and described it as a qualitative nominal variable with binary outcome recorded as yes and no.

The other information asked was on service delivery factors i.e., knowledge about nearest FP centers, number of operational days of the center, waiting time, available facilities, cost of services, counseling facility; socio-demographic factors such as age of the woman, education level of woman, area of residence, language spoken at home etc.; and information on contraceptive use.

The study questionnaire was based on the standard information from PDHS 2017-18 questionnaire and Service Provision Assessment survey form of the Measure. The interviews were conducted in the local language. Screening for the study participants was carried out through the records and registers of the lady health workers for the study catchment population. Data were collected from March 2021 to July 2021. Written informed consent was obtained from all eligible women who agreed to participate in the study. The ethical review committee of Aga Khan University gave ethical approval for conducting the study.

We used open epi software to estimate sample size of the study. The prevalence of various factors (distance (32.12%), counselling provided by healthcare providers (62.77%), and experienced side effects (80.29) for controls and cases were obtained from the literature search. We calculated sample size for all three factors mentioned and have selected range that

For peer review only - http://bmjopen.bmj.com/site/about/guidelines.xhtml

is covering all three factors. To account for non-response rate, we have increased sample size by 20% so, to determine an odds ratio of at least 2, by taking power of 80% and at significance of 5% with the ratio of 1:2 between cases and controls, a minimum of 141 cases and 282 controls were required for this study (4). Non-probability purposive sampling strategy was used to enroll study participants.

Data were analyzed using STATA version 14. Frequency and percentages are reported for qualitative variables such as area of residence, level of education, cost of services, and information related to side effects experienced by women. Mean and standard deviation are reported for continuous quantitative variables. We used logistic regression for both univariate and multivariable analysis to identify factors associated with continued use of a hormonal contraceptive method. A p-value of 0.25 was considered as criterion for variable to be significant at univariate level. Multicollinearity among all independent variables significant at univariate level was checked considering 0.80 as cut off. Logistic regression was used to identify factors associated with continuation of hormonal contraceptives while adjusting for other variables. A p-value of less than 0.05 was considered statistically significant. Biologically plausible interactions were assessed with significance of p value of less than 0.1. Odds ratios (OR) and their confidence intervals (CIs) were used for interpretation of results.

For peer review only

Results

Description of study population

We completed interviews on 141 cases and 282 controls. All the women in our study sample were married and were living with their husbands. Table 1 illustrates socio-demographic characteristics of continued users of hormonal method for at least last six months and current non-hormonal method users who used hormonal method and discontinued users. The mean age of women who were using hormonal method was 34.8 ± 5.9 years whereas mean age for

For peer review only - http://bmjopen.bmj.com/site/about/guidelines.xhtml

discontinued user was 31.5 ± 5.3 years. Among the continued users of hormonal methods, 31.9 % were residing in urban area and 68% were residing in rural areas of three selected union councils of Gilgit. Approximately, 77.3% of the discontinued users were living in rural areas and 22.7% were living in urban areas. Large number of women in both groups spoke Brushaski language (68% of the women among continued users and 72.3% among discontinued users). Less than 50% of the women could read newspaper in both the groups. Nearly ninety-three percent (92.9%) of the continued users and 95% of the discontinued users had access to television. Approximately, 44.6% of the continued users owned radio compared to 39% of the discontinued users.

Table1

Socio- demographic characteristics of continued and discontinued users of hormonal contraceptives.

**Variables**	**Continued users**<br>**n=141**<br>**n (%)**	**Discontinued users**<br>**n=282**<br>**n (%)**
**Age of women (years)**		
**Mean (SD)**	34.8 (5.9)	31.5 (5.3)
**Area of residence**		
**Rural**	45 (31.9)	64 (22.7)
**Urban**	96 (68.0)	218 (77.3)
**Education**		
**No formal education**	30 (21.2)	18 (6.3)
**Formal education**	111 (78.7)	264 (93.6)

For peer review only - http://bmjopen.bmj.com/site/about/guidelines.xhtml

**Language**		
**Brushaski**	96 (68.0)	204 (72.3)
**Others**	45 (31.9)	78 (27.6)
**Read news paper**		
**Yes**	58 (41.1)	120 (41.3)
**No**	83 (58.8)	162 (57.4)
**Watch TV**		
**Yes**	131 (92.9)	268 (95.0)
**No**	10 (7.0)	14 (4.9)
**listen radio**		
**Yes**	63 (44.6)	110 (39.0)
**No**	78 (55.3)	172 (60.9)

Table 2 shows Frequency distribution of biological factors among continued users and discontinued users of hormonal contraceptives. Among continued users, at the time of interview, 24.1% of the women were using pills, 2.1% of the women were using implant and 73.4% were using injectables for contraception. And among discontinued users, nearly 59.0%of the women were using traditional methods and 40.7% of the women were using condoms at the time of the interview. Before switching to non-hormonal methods these women were using pills (62.0%), injectables (37.5%) and implants (0.35%). Mean age at initiating contraceptive use among continued users was 26.2 years (SD 3.8) and among discontinued users 25.4 years (SD 3.1 years). Some difference was observed between two groups in number of living children at the time of initiating contraceptive use. Mean number

For peer review only - http://bmjopen.bmj.com/site/about/guidelines.xhtml

of children among continued users was 2 children (SD 1.4 years) and discontinued users had one child (standard deviation 1.2 years). Approximately sixty four percent (63.8%) of women among continued users mentioned taking a joint decision with husband regarding hormonal method use, whereas only 44% of discontinued users took joint decision for using a non-hormonal method. Interestingly 12.7% of continued users and 7.8% of discontinued users took self-decision for contraceptive use. Above 80% of women in both groups were given information regarding side effects and how to manage side effects of certain method by the providers. A large number of continued users (84.4) mentioned experiencing side effects.

Eighty-one percent of continued users and 79% discontinued users were availing contraceptive supplies from government source. Only 18.5% and 7.8% of continued and discontinued users were getting their supplies from private source respectively. Significant difference among both groups was seen in satisfaction level with the use of current method. Nearly 84% of continued users and 59.5% of discontinued users mentioned that they were satisfied with the current method they were using. Above 80% of continued users said that they get information related to family planning methods, family health counselling and contraceptive supplies from LHWs.

Table 2

Frequency distribution of biological factors among continued users and discontinued users of hormonal contraceptives.

**Variables**	**Continued users n=141 n (%)**	**Discontinued users n=282 n (%)**
**Age at initiating contraceptive use**		
**Mean (SD)**	26.2 (3.8)	25.4 (3.1)

For peer review only - http://bmjopen.bmj.com/site/about/guidelines.xhtml

**Number of living children at the time of initiating contraceptive use**		
**Mean (SD)**	2 (1.4)	1 (1.2)
**Decision maker of contraceptive use**		
**Self**	18 (12.7)	22 (7.8)
**Mainly Husband**	33 (23.4)	136 (48.2)
**Joint decision**	90 (63.8)	124 (43.9)
**Source of current method**		
**Government source**	114 (81.4)	90 (78.9)
**Private source**	26 (18.5)	24 (21.0)
**Information given regarding side effects**		
**Yes**	124 (87.9)	260 (87.9)
**No**	17 (12.0)	22 (7.8)
**Information given regarding other methods**		
**Yes**	**98 (69.5)**	**214 (75.8)**
**No**	**43 (30.5)**	**68 (24.1)**
**Experienced side effects**		
**Yes**	119 (84.4)	7 (2.4)
**No**	22 (15.6)	275 (97.5)

For peer review only - http://bmjopen.bmj.com/site/about/guidelines.xhtml

**Satisfied with current method**		
**Yes**	118 (83.6)	168 (59.5)
**No**	23 (16.3)	114 (40.4)
**Want more effective method**		
**Yes**	125 (44.3)	35 (24.8)
**No**	157 (55.6)	106 (75.1)
**Information given by LHW**		
**Mother and child health**	6 (4.2)	65 (23.0)
**Contraceptive supplies, Family planning and family health counseling**	120 (85.1)	209 (74.1)
**Others**	15 (10.6)	8 (2.8)

Factors related to service delivery

Table 3 illustrates factors related to service delivery among continued and discontinued users. All the women in our sample were aware of the nearest family planning centers of their area. Approximately eighty four percent (84.4%) of continued users and 73.0% of discontinued users had been to FP centers to avail services in last six months. Walking distance from home to nearest family planning center was measured subjectively by asking women if they could walk to access nearest center on foot or require transport to reach to the center. Approximately, 79.0% of the continued users mentioned that the family planning center is within walking distance while 21.2% needed a transport to reach to the nearest family planning center. Above thirty-eight (38.3%) % of the discontinued users were able to reach center on foot compared to 61% who required some kind of transport.

For peer review only - http://bmjopen.bmj.com/site/about/guidelines.xhtml

In both the groups, those women who did not visit FP clinics mentioned unsuitable timings of the FP center, far distance, non-availability of FP staff, cost of services, unavailability of desired method and transportation issues as some of the reasons for not availing the services.

Women were asked about their satisfaction from the services provided at home by the LHWs, 65.2% of women among continued users and 77.3% women among discontinued users were satisfied with home services.

Table 3

Frequency distribution of factors related to service deliver among continued and discontinued users.

**Variables**	**Continued users n=141 n (%)**	**Discontinued users n=282 n (%)**
**Ever been to FP centers to avail services**		
**Yes**	119 (84.4)	205 (72.7)
**No**	22 (15.6)	77 (27.3)
**Reasons for not going to FP centers**		
**Facility related issues**	4 (18.1)	27 (35.0)
**Services related issues**	13 (59.0)	34 (44.1)
**Transport related issues**	5 (22.7)	16 (20.7)
**Number of days services provided by center**		
**Mean (SD)**	6.0 (1)	5.7 (0.9)

For peer review only - http://bmjopen.bmj.com/site/about/guidelines.xhtml

For peer review only

**Routine fee charged by facility**		
**Yes**	133 (94.3)	277 (98.2)
**No**	8 (5.6)	5 (1.7)
**Presence of healthcare staff at center**		
**Yes**	105 (74.4)	163 (57.8)
**No**	36 (25.5)	119 (42.2)
**Counseling services at FP center**		
**Yes**	75 (53.1)	133 (47.1)
**No**	66 (46.8)	149 (52.8)
**Satisfied with services provided at home**		
**Yes**	92 (65.2)	218 (77.3)
**No**	49 (34.7)	64 (22.7)
**Reasons for dissatisfaction**		
**Did not like method**	12 (24.4)	10 (15.6)
**Other (have more side-effects, provide counselling only)**	37 (75.5)	54 (84.3)
**Distance from home to FP center**		
**Near to my house at walking distance**	111 (78.7)	108 (38.3)
**Far need transport**	30 (21.2)	174 (61.7)
**Cost of round trip in USD**		
**No cost**	110 (80.1)	112 (39.7)
**≤0.4**	5 (1.4)	55 (19.5)
**≤2**	17 (12.0)	78 (27.6)

For peer review only - http://bmjopen.bmj.com/site/about/guidelines.xhtml

**>2**	9 (6.3)	37 (13.1)

On univariate analysis, higher mean age of women among continued users, residing in rural area, no formal education, joint decision making regarding contraceptive use, information given by lady health workers, visiting FP centers, presence of healthcare staff at the center, family planning counselling at centers, living closer to family planning centers were significantly associated with hormonal contraceptive continuation at p level of less than 0.05.

On multivariable analysis after adjusting for effect of other variables in the model walking distance to a family planning center (AOR=6.33, 95% CI=3.74-10.71) visiting family planning center (AOR=1.86, 95% CI=1.07-3.45), older age of the women (AOR=1.07, CI=1.02-1.12), having formal education (AOR 0.27, 95% CI=0.13-0.47) were independently associated with continuation of hormonal contraceptives as shown in Table 4.

Table 4 Adjusted odds ratio (95% confidence interval) for the factors associated with continuation of hormonal contraceptives among continued and discontinued users in Gilgit, Pakistan.

**Variables**	**Continued users n=141 n (%)**	**Discontinued users n=298 n (%)**	**adjusted odds ratio (95% C.I)**
**Distance from home to FP center**			
**need transport**	30 (21.2)	174 (61.7)	1
**at walking distance**	111 (78.7)	108 (38.3)	6.4 (3.70-11.19)

For peer review only

For peer review only - http://bmjopen.bmj.com/site/about/guidelines.xhtml

**Age of women (years)**			
**Mean (SD)**	34.8 (5.9)	31.5 (5.3)	1.06 (1.01-1.11)
**Education**			
**No formal education**	30 (21.2)	18 (6.3)	1
**Formal Education**	111 (78.7)	264 (93.6)	0.27 (0.13-0.47)
**Satisfied with current method**			
**No**	23 (16.3)	114 (40.4)	1
**Yes**	118 (83.6)	168 (59.5)	3.64 (2.06-6.44)
**Visits FP center**			
**No**	22 (15.6)	77 (27.3)	1
**Yes**	119 (84.4)	205 (72.7)	1.86 (1.07-3.45)

For peer review only - http://bmjopen.bmj.com/site/about/guidelines.xhtml

Discussion

Our study results indicate that both service delivery and individual factors are associated with continuation of hormonal contraceptive use for at least six months for married women of Gilgit Baltistan. In our study sample, significant service delivery related factors included proximity of the center to the area of residence, information given about side effects of the method by FP center staff, satisfaction with current method and visiting nearest family planning center. Other significant individual factors were age and education status of the women.

Our results are in concordance with other studies (14). Proximity to the health facilities and facilities providing wide range of contraceptives increases of the use of contraceptive methods, Physical access to family planning services and other maternal and neonatal health facilities is mostly determined by distance to travel. (15,16,17,18). Access to family planning centers within 1.9 km and that provide four or more methods increase contraceptive use and as distance increases from two kilometers irrespective of number of methods provided by the facility the contraceptive use starts to decline (19). Previous studies from Pakistan also found that proximity to functional family planning centers is strongly associated with contraceptive use (16,18). Our study also suggests that walking distance from home to family planning centers matters for the use of a contraceptive method.

In our study sample, women who had ever been to formal school were less likely to use a hormonal contraceptive compared to those who had never been to formal school, this finding contrasted with existing literature. Conventionally, women with no formal education are less likely to practice modern contraceptives compared with some formal education (20) and women's education was strong predictor of contraceptive use and women who had attained secondary or more education were twice likely to avail contraceptive services (21). We believe, in our study, closer distance and easy access to contraceptive method of choice has made

For peer review only - http://bmjopen.bmj.com/site/about/guidelines.xhtml

education less important as a factor for women to use of a contraceptive method. In context of Gilgit, living near to FP centers and door to door visits by LHWs has made women of the region more aware about the contraceptive methods irrespective of education status.

Our study found that satisfaction with the method in current use is one of the significant reason to continue hormonal method of contraception. Client's satisfaction with a method makes users compliant to use and becomes a main factor for its continuation. In our study, all the women said that they have been visited by a lady health worker in last six months and they had received contraceptive counselling (side-effects, how to manage side-effects and seek treatment in case of experiencing side-effects) and referral. Women who have been visited by a lady health worker and discussed family planning in the last twelve months is positively associated with continuing long-acting reversible contraceptives (24), similarly, women in Malawi who had been visited by lady health workers were more likely to continue injectables compared to other contraceptives (19). Hence lady health workers are critical source of information for introducing women to hormonal contraceptives (25). LHW's in Gilgit district provide door to door contraceptive counselling and make women aware of possible side-effects of a contraceptive method and refer them and counsel them about ways to manage and seek treatment for the side-effects. We found positive association of contraceptive use with age. Women aged 20-44 prefer short acting compared to women aged 15-19 who prefer long-acting contraceptives (26). As family of a woman completes, she desires to limit pregnancies and hence contraceptive compliance increases as age of the women increases (26).

Our study had several strengths. Firstly, recruitment of the study participants was based on identification from lady health workers records as they do frequent visits and keep their records updated and we reconfirmed from the women before recruiting. This was a robust method leading to decrease chance of misclassification of the outcome status of the participants. Moreover, case to control ratio was kept 1:2 which is more likely to represent

For peer review only - http://bmjopen.bmj.com/site/about/guidelines.xhtml

experience of general population and similar data collection tool was used to collect data from cases and controls. Major limitation of the study was the subjective assessment of walking distance due to unavailability of internet access in all union councils of district Gilgit. However, those women who never used transport to reach FP is supportive of giving an affirmation of the proximity of FP centers from their homes. Furthermore, this study is generalizable to areas that are geographically like district Gilgit.

Conclusion and recommendations

In Gilgit, continuation of hormonal method use was associated with easy access to family planning centers, satisfaction with current method use and visits to FP center. In addition, continuation was also seen in women with no formal education. Women who were using hormonal method continuously were three years older than discontinued users. Despite experiencing side-effects women who were using hormonal contraceptives continued to use method of their choice for at least six months. Our study findings recommends that family planning centers should be closer to the residential areas to make access of women easier. Moreover, appropriate counselling, reassurance and response to side-effects are key for continuation of hormonal method. Counselling regarding managing side-effects by lady health workers at home and by health care providers at centers should be ensured. In future, a cohort study to observe behavior of women for using, continuing, switching over and discontinuing hormonal method would add to knowledge regarding continuous use of hormonal methods.

Dissemination Plan

The findings of this study will be presented at important national and international conferences and published in peer-reviewed scientific journals.

Ethics approval and consent to participate

For peer review only

For peer review only - http://bmjopen.bmj.com/site/about/guidelines.xhtml

This study was approved by Ethical Review Committee (ERC) Aga Khan University (2022-6023-21033). All methods were performed in accordance with the ethical guidelines and regulations of ERC Aga Khan University. Informed consent was taken from each respondent before data collection of the study. Participants were assured of confidentiality and privacy.

Consent of Publication

Not applicable

Availability of data and materials

The dataset used during the current study is available from the corresponding author on reasonable request.

Competing Interests

The authors have declared that no competing interests exists.

Funding

Funding from HRP Alliance, part of UNDP-UNFPA-UNICEF-WHO-World Bank special program of research, development, and Research Training in Human Reproduction (HRP), a cosponsored program executed by The World Health Organization (WHO). The main funders HRP had no role in study design, data collection and analysis.

Authors contribution

Fazila Bibi

Roles: Conceptualization, Data Curation, Data Analysis, investigation, Methodology, Software, Original draft, writing.

Affiliation: Center of Excellence for Trauma and Emergencies, Aga Khan University, Karachi, Pakistan

Sarah Saleem

Roles: Conceptualization, Methodology, Supervision, Writing-Review and Editing.

For peer review only - http://bmjopen.bmj.com/site/about/guidelines.xhtml

Affiliation: Department of Community Health Sciences, Aga Khan University, Karachi, Pakistan

Shiyam Sunder

Roles: Methodology, Writing-Review and Editing

Affiliation: Department of Community Health Sciences, Aga Khan University, Karachi, Pakistan

Shafquat Rozi

Roles: Data Analysis

Affiliation: Department of Community Health Sciences, Aga Khan University, Karachi, Pakistan

For peer review only

Acknowledgements

Fazila Bibi received funding from HRP Alliance, part of UNDP-UNFPA-UNICEF-WHO-World Bank special program of research, development, and Research Training in Human Reproduction (HRP), a cosponsored program executed by The World Health Organization (WHO) to complete her studies. This article represents the views of the named authors only and does not represent the views of World Health Organization.

References

1. Mustafa G, Azmat SK, Hameed W, Ali S, Ishaque M, Hussain W, et al. Family Planning Knowledge, Attitudes, and Practices among Married Men and Women in Rural Areas of Pakistan: Findings from a Qualitative Need Assessment Study. Int J Reprod Med. 2015; 2015:190520.

2. Ontiri S, Mutea L, Naanyu V, Kabue M, Biesma R, Stekelenburg J. A qualitative exploration of contraceptive use and discontinuation among women with an unmet need for modern contraception in Kenya. Reprod Health. 2021;18(1):33.

3. Affairs UNDoEaS. Contraceptive use by method. 2019.

For peer review only - http://bmjopen.bmj.com/site/about/guidelines.xhtml

https://www.un.org/development/desa/pd/sites/www.un.org.development.desa.pd/files/files/documents/2020/Jan/un_2019_contraceptiveusebymethod_databooklet.pdf

4. Thobani R, Jessani S, Azam I, Reza S, Sami N, Rozi S, et al. Factors associated with the discontinuation of modern methods of contraception in the low-income areas of Sukh Initiative Karachi: A community-based case control study. PLoS One. 2019;14(7):e0218952.

5. Bank W. Family planning in South Asia: Improving equity of access [Internet]2012. [cited 2022]. Available from: https://blogs.worldbank.org/health/family-planning-in-south-asia-improving-equity-of-access.

6. Pakistan U. Multiple Indicator Cluster Survey 2016-17 Report and Key Findings - Gilgit Baltistan. 2017.

7. Okigbo CC, Speizer IS, Domino ME, Curtis SL. A Multilevel Logit Estimation of Factors Associated with Modern Contraception in Urban Nigeria. World Med Health Policy. 2017;9(1):65-88.

8. Todd N, Black A. Contraception for Adolescents. J Clin Res Pediatr Endocrinol. 2020;12(Suppl 1):28-40.

9. Hong R, Montana L, Mishra V. Family planning services quality as a determinant of use of IUD in Egypt. BMC Health Serv Res. 2006; 6:79.

10. Kungu W, Agwanda A, Khasakhala A. Prevalence of and factors associated with contraceptive discontinuation in Kenya. Afr J Prim Health Care Fam Med. 2022;14(1):e1-e11.

11. Sadat-Hashemi SM, Ghorbani R, Majdabadi HA, Farahani FK. Factors associated with contraceptive use in Tehran, Iran. Eur J Contracept Reprod Health Care. 2007;12(2):148-53.

12. Hall KS, Castaño PM, Westhoff CL. The influence of oral contraceptive knowledge on oral contraceptive continuation among young women. J Womens Health (Larchmt). 2014;23(7):596-601.

13. UNFPA. Mapping of Public and Private Sector for Family Planning and Reproductive Health Services in Selected Districts in AJK and GB - Report. 2020.

14. Shiferaw, S., Spigt, M., Seme, A., Amogne, A., Skrøvseth, S., Desta, S., Radloff, S., Tsui, A. and GeertJan, D., 2017. Does proximity of women to facilities with better choice of contraceptives affect their contraceptive utilization in rural Ethiopia. *PLOS ONE*, 12(11), p.e0187311.

For peer review only - http://bmjopen.bmj.com/site/about/guidelines.xhtml

15. Okwaraji YB, Webb EL, Edmond KM. Barriers in physical access to maternal health services in rural Ethiopia. BMC Health Serv Res. 2015; 15:493.

16. Aparna Sundaram RH, Zeba Sathar, Sabahat Hussain, Weissman EPaE. Adding It Up: Costs and Benefits of Meeting the Contraceptive and Maternal and Newborn Health Needs of Women in Pakistan. Population Council. 2019

17. Sumera Aziz Ali SS, Neelofar Sami, Mir Shabbar, Muhammad Ahmed. Geographic Access to Working Family Planning Centers and Unintended Pregnancies among Married Women: A Community Based Nested Case Control Study. Open Journal of Epidemiology. 2016.

18. Sumera Aziz Ali ZF. Predictors of modern contraceptive usage among women in rural Pakistan physical access to services still a major barrier. EC Gynecology. 2017.

19. Digitale J, Psaki S, Soler-Hampejsek E, Mensch BS. Correlates of Contraceptive Use and Health Facility Choice among Young Women in Malawi. Ann Am Acad Pol Soc Sci. 2017;669(1):93-124.

20. Aviisah PA, Dery S, Atsu BK, Yawson A, Alotaibi RM, Rezk HR, et al. Modern contraceptive use among women of reproductive age in Ghana: analysis of the 2003-2014 Ghana Demographic and Health Surveys. BMC Womens Health. 2018;18(1):141.

21. Rios-Zertuche D, Blanco LC, Zúñiga-Brenes P, Palmisano EB, Colombara DV, Mokdad AH, et al. Contraceptive knowledge and use among women living in the poorest areas of five Mesoamerican countries. Contraception. 2017;95(6):549-57.

22. Westhoff CL, Heartwell S, Edwards S, Zieman M, Stuart G, Cwiak C, et al. Oral contraceptive discontinuation: do side effects matter? Am J Obstet Gynecol. 2007;196(4): 412.e1-6; discussion. e6-7.

23. Culwell K, Hillard, P, Glob. Patient education and contraceptive compliance. 2008

24. Hossain S, Sripad P, Zieman B, Roy S, Kennedy S, Hossain I, et al. Measuring quality of care at the community level using the contraceptive method information index plus and client reported experience metrics in Bangladesh. J Glob Health. 2021; 11:07007.

25. Nisar S, Khan M, Nisar U. Impact Of Lady Health Workers On The Contraceptive Prevalence Rate In District Mardan. J Ayub Med Coll Abbottabad. 2020;32(1):104-10.

26. Tibaijuka L, Odongo R, Welikhe E, Mukisa W, Kugonza L, Busingye I, et al. Factors influencing use of long-acting versus short-acting contraceptive methods

For peer review only - http://bmjopen.bmj.com/site/about/guidelines.xhtml

among reproductive-age women in a resource-limited setting. BMC Womens Health. 2017;17(1):25.

For peer review only

For peer review only - http://bmjopen.bmj.com/site/about/guidelines.xhtml

BMJ Open

Factors associated with continuation of hormonal contraceptives among married women of reproductive age in Gilgit- Pakistan: a community-based case control study

Journal:	*BMJ Open*
Manuscript ID	bmjopen-2023-075490.R1
Article Type:	Original research
Date Submitted by the Author:	13-Sep-2023
Complete List of Authors:	Bibi, Fazila; The Aga Khan University, Center of Excellence for Trauma and Emergencies<br>Saleem, Sarah; Aga Khan University, Community Health Sciences<br>Tikmani, Shiyam; Aga Khan University, Community Health Sciences<br>Rozi, Shafquat; Aga Khan University, Community Health Sciences
**Primary Subject Heading**:	Obstetrics and gynaecology
Secondary Subject Heading:	Sexual health, Public health
Keywords:	SEXUAL MEDICINE, REPRODUCTIVE MEDICINE, GYNAECOLOGY, Health Services Accessibility, Reproductive medicine < GYNAECOLOGY

SCHOLARONE™
Manuscripts

I, the Submitting Author has the right to grant and does grant on behalf of all authors of the Work (as defined in the below author licence), an exclusive licence and/or a non-exclusive licence for contributions from authors who are: i) UK Crown employees; ii) where BMJ has agreed a CC-BY licence shall apply, and/or iii) in accordance with the terms applicable for US Federal Government officers or employees acting as part of their official duties; on a worldwide, perpetual, irrevocable, royalty-free basis to BMJ Publishing Group Ltd ("BMJ") its licensees and where the relevant Journal is co-owned by BMJ to the co-owners of the Journal, to publish the Work in this journal and any other BMJ products and to exploit all rights, as set out in our licence.

The Submitting Author accepts and understands that any supply made under these terms is made by BMJ to the Submitting Author unless you are acting as an employee on behalf of your employer or a postgraduate student of an affiliated institution which is paying any applicable article publishing charge ("APC") for Open Access articles. Where the Submitting Author wishes to make the Work available on an Open Access basis (and intends to pay the relevant APC), the terms of reuse of such Open Access shall be governed by a Creative Commons licence – details of these licences and which Creative Commons licence will apply to this Work are set out in our licence referred to above.

Other than as permitted in any relevant BMJ Author's Self Archiving Policies, I confirm this Work has not been accepted for publication elsewhere, is not being considered for publication elsewhere and does not duplicate material already published. I confirm all authors consent to publication of this Work and authorise the granting of this licence.

For peer review only

Factors associated with continuation of hormonal contraceptives among married women of reproductive age in Gilgit- Pakistan: a community-based case control study.

Fazila Bibi[1*], Sarah Saleem[2], Shiyam Sunder Tikmani[3], Shafquat Rozi[4]

[1]Center of Excellence for Trauma and Emergencies, Aga Khan University, Karachi, Pakistan

[2]Community Health Sciences, Aga Khan University, Karachi, Pakistan

[3]Community Health Sciences, Aga Khan University, Karachi, Pakistan

[4]Community Health Sciences, Aga Khan University, Karachi, Pakistan

Author information:

Fazila Bibi (FB)- Fazila.sahibjan@aku.edu

Sarah Saleem (SS)- sarah.saleem@aku.edu

Shiyam Sunder Tikmani (SST) Shiyam.sunder@aku.edu

Shafquat Rozi (SR)- Shafquat.rozi@aku.edu

Correspondence to: Fazila.sahibjan@aku.edu

Centre of Excellence for Trauma and Emergencies, Aga Khan University, Karachi,

Address: Stadium Road, P.O. Box-3500, Karachi (74800), Pakistan.

Tel: 92-21-3486-4837

Fax: 92-21-34932095

For peer review only

ABSTRACT

Objective

This study aimed to determine the factors associated with continuation of hormonal contraceptive methods among married women of Gilgit, Pakistan at least six months after their initiation.

Design

Unmatched case control study.

Setting

Community settings of district Gilgit, Pakistan from April 1, 2021, to July 30, 2021.

Participants

The cases were married women of reproductive age who at the time of interview were using a hormonal method of contraception for at least last six months continuously, and controls were married women of reproductive age who had used hormonal method in the past and currently were using a non-hormonal method for at least last six months.

Primary and Secondary outcome measures

OR for continuation of hormonal contraceptive.

Results

The factors significantly associated with continuous use of hormonal contraceptive methods for our sample from Gilgit were family planning center at the walking distance from home (AOR 6.33, 95% CI, 3.74-10.71), satisfaction with current method in use (AOR 3.64 95% CI, 2.06-6.44), visits to the family planning center to avail services (AOR 1.86 95% CI, 1.07-3.45) and relatively older age of women (AOR 1.07, 95% C1, 1.02-1.12). In addition, women with formal education (AOR 0.27 95% CI, 0.12-0.6), were less likely to use a modern contraceptive method.

Conclusion

For peer review only - http://bmjopen.bmj.com/site/about/guidelines.xhtml

Continuation of a hormonal method use was associated with easy access to family planning centers, satisfaction with the current method use and frequent visits to the family planning centers. Continuation of hormonal method use was also seen in women with low education status. The importance of the presence of family planning centers near residential areas cannot be emphasized more. This not only provides easy access to FP methods, but also reassures women for continuation of modern methods when they face any unpleasant effects while using these.

Strengths and limitations of this study

- Case to control ratio was kept 1:2 which is more likely to represent experience of general population.
- Subjective assessment of walking distance due to unavailability of internet access in all union councils of district Gilgit.
- This study is generalizable to areas that are geographically similar to district Gilgit.
- Data was collection was done during Covid19 times; women might have discontinued going to family planning centers for hormonal contraceptives due to fear of getting infection and this might have affected our results.

For peer review only

Introduction

Access to safe and effective contraceptives is the right of every woman and man irrespective of their religion, or socio-economic status, region, or country, so that, they can make an informed choice of their preferred type of method they would like to use (1). The trends of contraceptive use, its discontinuation and switching a method are significant identifiers of how well family planning programs are fulfilling the needs of family planning of reproductive age women and couples in a country (2). Worldwide 922 million women of reproductive age use any form of contraceptives (3), however, there are still 12 percent of the women who want to avoid pregnancy and are not using any method of contraception (4). High fertility rates and low contraceptive prevalence (CPR) rates are the main elements of exponential population growth in South Asian countries (5). In Pakistan, the high fertility rate and low use of contraceptives are contributing factors to poor reproductive health indicators for maternal mortality. Furthermore, discontinuation of contraception has been found to be one of the major reasons of induced abortions in Pakistan. Multi-indicator cluster survey of Pakistan (MICS 2016-17) reported that in Gilgit Baltistan (GB) 38.1% of married women of reproductive age had access to contraception, of these 32.1% use one of the modern contraceptive methods (6).

Contraceptive continuation is the process of uninterrupted usage of contraceptive methods for at least six months. Hormonal methods use hormones to regulate ovulation and prevent pregnancy that include injectable birth controls, contraceptive skin patch, vaginal ring and oral contraceptive pills. Factors associated with contraceptive continuation are both methods related such as efficacy of the method, frequency of doses, and client- related such as age, education level, parity etc. (7,8). Distance of the family planning center from home and availability of different methods at the center are additional important factors contributing to continuity in use of a method. Presence of healthcare staff at the center, level of education,

For peer review only

age, number of living children and area of residence were all found to be associated with contraceptive continuation (9,10,11,12).

Information regarding predictors of continuation of hormonal contraceptives is scarce from Pakistan and information related to continuation of any contraceptive method is mostly unavailable in Pakistan. Findings from this study would help in improving contraceptive prevalence rate and decreasing discontinuation of hormonal methods which are more effective compared to non-hormonal method. Moreover, promoting factors that are associated with the continuation of hormonal method will help to avoid untimed and unwanted pregnancies hence it will improve maternal health.

Gilgit Baltistan is situated in Northern part of Pakistan which covers an area of over 72,971 KM^2 and is highly hilly and mountainous. Gilgit is the capital and largest district with estimated population of 216,760 (13). Seventy-nine percent of family planning centers with trained staff in Gilgit are in the main town while remaining are in other union councils with 4-29 % trained staff (13) as shown in figure1.

This suggests that all specialized facilities are centered in Gilgit town and women from other union councils must travel to get specialized services and the average distance from Gilgit town to the nearest health facility is 2.7 kilometers which is far less than other union councils that are 18-24 kilometers away from the nearest health facility (13). Considering the high efficacy of the hormonal methods to prevent pregnancy, we were interested in identifying region-specific (Gilgit) factors that were associated with the continuation of hormonal contraceptives.

Methods

We used a community-based unmatched case control study design, with ratio of cases to control as 1:2. Cases were married women of reproductive age who were currently using a hormonal method of contraception for at least last six months, and controls were married

For peer review only

women of reproductive age who had ever used a hormonal method but now are using a non-hormonal method at least for last six months, This was to know why women switched from hormonal to non-hormonal method and to make groups comparable we selected control group with history of hormonal method use. We collected data from the catchment population of LHWs of 3 union councils Gilgit town, Danyore, and Nomal. Hormonal method users were sexually active women who were using one of the hormonal contraceptive methods i.e., pills, injectables or implants for at least last six months without disruption at the time of enrolment in the study and Controls were sexually active married women of reproductive age, with history of using a hormonal method but stopped it before six months after initiating it and switched to a non-hormonal contraceptive method for at least last six months. We excluded women using intrauterine contraceptive device (IUCD), as there are of two types (hormonal and non-hormonal) methods and women might not know the type of IUCD she was using. Women who were using permanent method of contraception were also excluded. Data were collected from March 2021 to July 2021. Written informed consent was obtained from all eligible women who agreed to participate in the study. This study was approved by ethical review committee of Aga Khan University.

To account for non-response rate, sample size was increased by 20% so, to determine an odds ratio of at least 2, by taking power of 80% and at significance of 5% with the ratio of 1:2 between cases and controls, a minimum of 141 cases and 282 controls were required for this study (4).

Data were analyzed using Stata version 14. Frequency and percentages are reported for qualitative variables. Mean and standard deviation are reported for continuous quantitative variables. We used logistic regression for both univariate (simple logistic regression) and multivariable analysis to identify factors associated with continued use of a hormonal contraceptive method. Multicollinearity among all independent variables significant at

For peer review only - http://bmjopen.bmj.com/site/about/guidelines.xhtml

univariate level was checked considering 0.80 as cut off for Pearson correlation (for continuous variables), Crammers V to check multicollinearity among categorical variables.

Patient and Public Involvement

No patient and public involvement in current study.

Results

<u>Description of study population</u>

<u>Table1</u>

Socio- demographic characteristics of continued and discontinued users of hormonal contraceptives.

Variables	Continued users<br>n=141<br>n (%)	Discontinued users<br>n=282<br>n (%)
**Age of women (years)**		
**Mean (SD)**	34.8 (5.9)	31.5 (5.3)
**Area of residence**		
**Rural**	45 (31.9)	64 (22.7)
**Urban**	96 (68.0)	218 (77.3)
**Education**		
**No formal education**	30 (21.2)	18 (6.3)
**Formal education**	111 (78.7)	264 (93.6)
**Language**		

For peer review only - http://bmjopen.bmj.com/site/about/guidelines.xhtml

For peer review only

**Brushaski**	96 (68.0)	204 (72.3)
**Others**	45 (31.9)	78 (27.6)

We completed interviews on 141 cases and 282 controls. Table 1 illustrates socio-demographic characteristics of continued users of hormonal method for at least last six months and current non-hormonal method users who used hormonal method and discontinued users. The mean age of women who were using hormonal method was 34.8 ± 5.9 years whereas mean age for discontinued user was 31.5 ± 5.3 years.

Table 2

Frequency distribution of biological factors among continued users and discontinued users of hormonal contraceptives.

**Variables**	**Continued users n=141 n (%)**	**Discontinued users n=282 n (%)**
**Age at initiating contraceptive use**		
**Mean (SD)**	26.2 (3.8)	25.4 (3.1)
**Number of living children at the time of initiating contraceptive use**		
**Mean (SD)**	2 (1.4)	1 (1.2)
**Decision maker of contraceptive use**		
**Self**	18 (12.7)	22 (7.8)
**Mainly Husband**	33 (23.4)	136 (48.2)
**Joint decision**	90 (63.8)	124 (43.9)

For peer review only - http://bmjopen.bmj.com/site/about/guidelines.xhtml

For peer review only

**Source of current method**		
**Government source**	114 (81.4)	90 (78.9)
**Private source**	26 (18.5)	24 (21.0)
**Information given regarding side effects**		
**Yes**	124 (87.9)	260 (87.9)
**No**	17 (12.0)	22 (7.8)
**Information given regarding other methods**		
**Yes**	**98 (69.5)**	**214 (75.8)**
**No**	**43 (30.5)**	**68 (24.1)**
**Experienced side effects**		
**Yes**	119 (84.4)	7 (2.4)
**No**	22 (15.6)	275 (97.5)
**Satisfied with current method**		
**Yes**	118 (83.6)	168 (59.5)
**No**	23 (16.3)	114 (40.4)
**Want more effective method**		
**Yes**	125 (44.3)	35 (24.8)
**No**	157 (55.6)	106 (75.1)

For peer review only

For peer review only - http://bmjopen.bmj.com/site/about/guidelines.xhtml

**Information given by LHW**		
**Mother and child health**	6 (4.2)	65 (23.0)
**Contraceptive supplies, Family planning and family health counseling**	120 (85.1)	209 (74.1)
**Others**	15 (10.6)	8 (2.8)

Table 2 shows Frequency distribution of biological factors among continued users and discontinued users of hormonal contraceptives. Among continued users, at the time of interview, 24.1% of the women were using pills, 2.1% of the women were using implant and 73.4% were using injectables for contraception. And among discontinued users, nearly 59.0%of the women were using traditional methods and 40.7% of the women were using condoms at the time of the interview. Before switching to non-hormonal methods these women were using pills (62.0%), injectables (37.5%) and implants (0.35%). Mean age at initiating contraceptive use among continued users was 26.2 years (SD 3.8) and among discontinued users 25.4 years (SD 3.1 years). Some difference was observed between two groups in number of living children at the time of initiating contraceptive use. Mean number of children among continued users was 2 children (SD 1.4 years) and discontinued users had one child (SD 1.2 years).

Factors related to service delivery.

Table 3

Frequency distribution of factors related to service deliver among continued and discontinued users.

**Variables**	**Continued users n=141 n (%)**	**Discontinued users n=282 n (%)**

For peer review only - http://bmjopen.bmj.com/site/about/guidelines.xhtml

**Ever been to FP centers to avail services**		
**Yes**	119 (84.4)	205 (72.7)
**No**	22 (15.6)	77 (27.3)
**Reasons for not going to FP centers**		
**Facility related issues**	4 (18.1)	27 (35.0)
**Services related issues**	13 (59.0)	34 (44.1)
**Transport related issues**	5 (22.7)	16 (20.7)
**Number of days services provided by center**		
**Mean (SD)**	6.0 (1)	5.7 (0.9)
**Routine fee charged by facility**		
**Yes**	133 (94.3)	277 (98.2)
**No**	8 (5.6)	5 (1.7)
**Presence of healthcare staff at center**		
**Yes**	105 (74.4)	163 (57.8)
**No**	36 (25.5)	119 (42.2)
**Counseling services at FP center**		
**Yes**	75 (53.1)	133 (47.1)
**No**	66 (46.8)	149 (52.8)
**Satisfied with services provided at home**		
**Yes**	92 (65.2)	218 (77.3)
**No**	49 (34.7)	64 (22.7)

For peer review only - http://bmjopen.bmj.com/site/about/guidelines.xhtml

**Reasons for dissatisfaction**		
**Did not like method**	12 (24.4)	10 (15.6)
**Other (have more side-effects, provide counselling only)**	37 (75.5)	54 (84.3)
**Distance from home to FP center**		
**Near to my house at walking distance**	111 (78.7)	108 (38.3)
**Far need transport**	30 (21.2)	174 (61.7)
**Cost of round trip in USD**		
**No cost**	110 (80.1)	112 (39.7)
**≤0.4**	5 (1.4)	55 (19.5)
**≤2**	17 (12.0)	78 (27.6)
**>2**	9 (6.3)	37 (13.1)

Table 3 illustrates factors related to service delivery among continued and discontinued users. All the women in our sample were aware of the nearest family planning centers of their area. Approximately eighty four percent (84.4%) of continued users and 73.0% of discontinued users had been to FP centers to avail services in last six months. Walking distance from home to nearest family planning center was measured subjectively by asking women if they could walk to access nearest center on foot or require transport to reach to the center. Approximately, 79.0% of the continued users mentioned that the family planning center is within walking distance while 21.2% needed a transport to reach to the nearest family

For peer review only - http://bmjopen.bmj.com/site/about/guidelines.xhtml

planning center. Above thirty-eight (38.3%) % of the discontinued users were able to reach center on foot compared to 61% who required transport.

In both the groups, those women who did not visit FP clinics mentioned unsuitable timings of the FP center, far distance, non-availability of FP staff, cost of services, unavailability of desired method and transportation issues as some of the reasons for not availing the services.

Women were asked about their satisfaction from the services provided at home by the LHWs, 65.2% of women among continued users and 77.3% women among discontinued users were satisfied with home services.

Table 4 Adjusted odds ratio (95% confidence interval) for the factors associated with continuation of hormonal contraceptives among continued and discontinued users in Gilgit, Pakistan.

Variables	Continued users n=141 n (%)	Discontinued users n=298 n (%)	adjusted odds ratio (95% C.I)
**Distance from home to FP center**			
**need transport**	30 (21.2)	174 (61.7)	1
**at walking distance**	111 (78.7)	108 (38.3)	6.4 (3.70-11.19)
**Age of women (years)**			
**Mean (SD)**	34.8 (5.9)	31.5 (5.3)	1.06 (1.01-1.11)
**Education**			

For peer review only

**No formal education**	30 (21.2)	18 (6.3)	1
**Formal Education**	111 (78.7)	264 (93.6)	0.27 (0.13-0.47)
**Satisfied with current method**			
**No**	23 (16.3)	114 (40.4)	1
**Yes**	118 (83.6)	168 (59.5)	3.64 (2.06-6.44)
**Visits FP center**			
**No**	22 (15.6)	77 (27.3)	1
**Yes**	119 (84.4)	205 (72.7)	1.86 (1.07-3.45)

*Only statistically significant variables are shown in table 4

On multivariable analysis after adjusting for effect of other variables in the model walking distance to a family planning center (AOR=6.33, 95% CI=3.74-10.71) visiting family planning center (AOR=1.86, 95% CI=1.07-3.45), older age of the women (AOR=1.07, CI=1.02-1.12), having formal education (AOR 0.27, 95% CI=0.13-0.47) were independently associated with continuation of hormonal contraceptives as shown in Table 4.

Discussion

Our study results indicate that both service delivery and individual factors are associated with continuation of hormonal contraceptive use for at least six months for married women of Gilgit Baltistan. In our study sample, significant service delivery related factors included proximity of the center to the area of residence, information given about side effects of the

For peer review only
For peer review only - http://bmjopen.bmj.com/site/about/guidelines.xhtml

method by FP center staff, satisfaction with current method and visiting nearest family planning center. Other significant individual factors were age and education status of the women.

Our results are in concordance with other studies (14). Proximity to the health facilities and facilities providing wide range of contraceptives increases of the use of contraceptive methods, Physical access to family planning services and other maternal and neonatal health facilities is mostly determined by distance to travel. (15,16,17,18). Access to family planning centers within 1.9 km and that provide four or more methods increase contraceptive use and as distance increases from two kilometers irrespective of number of methods provided by the facility the contraceptive use starts to decline (19). Previous studies from Pakistan also found that proximity to functional family planning centers is strongly associated with contraceptive use (16,18). Our study also suggests that walking distance from home to family planning centers matters for the use of a contraceptive method.

In our study sample, women who had ever been to formal school were less likely to use a hormonal contraceptive compared to those who had never been to formal school. Conventionally, women with no formal education are less likely to practice modern contraceptives compared with some formal education (20) and women's education was strong predictor of contraceptive use and women who had attained secondary or more education were twice likely to avail contraceptive services (21). However, there can be religious reasons for preferring non-invasive and non-chemical methods. Upper class women face more facilitating conditions for traditional contraceptive use such as better husband wife communication, which is so important for effective birth control and more importantly, they may be better able to deal with contraceptive failure because they have capacity to afford an additional child and they also have easy access to abortion (22). We believe, in our study, closer distance and easy access to contraceptive method of choice has made education less important as a factor for women to

For peer review only - http://bmjopen.bmj.com/site/about/guidelines.xhtml

use of a contraceptive method. In context of Gilgit, living near to FP centers and door to door visits by LHWs has made women of the region more aware about the contraceptive methods irrespective of education status.

Our study found that satisfaction with the method in current use is one of the significant reasons to continue hormonal method of contraception. Client's satisfaction with a method makes users compliant to use and becomes a main factor for its continuation. In our study, all the women said that they have been visited by a lady health worker in last six months and they had received contraceptive counselling (side-effects, how to manage side-effects and seek treatment in case of experiencing side-effects) and referral. Women who have been visited by a lady health worker and discussed family planning in the last twelve months is positively associated with continuing long-acting reversible contraceptives (23), similarly, women in Malawi who had been visited by lady health workers were more likely to continue injectables compared to other contraceptives (19). Hence lady health workers are critical source of information for introducing women to hormonal contraceptives (24). LHW's in Gilgit district provide door to door contraceptive counselling and make women aware of possible side-effects of a contraceptive method and refer them and counsel them about ways to manage and seek treatment for the side-effects. We found positive association of contraceptive use with age. Women aged 20-44 prefer short acting compared to women aged 15-19 who prefer long-acting contraceptives (25,27). As family of a woman completes, she desires to limit pregnancies and hence contraceptive compliance increases as age of the women increases (25,26).

Conclusion and recommendations

In Gilgit, continuation of hormonal method use was associated with easy access to family planning centers, satisfaction with current method use and visits to FP center. Our study findings recommends that family planning centers should be closer to the residential areas to

For peer review only - http://bmjopen.bmj.com/site/about/guidelines.xhtml

make access of women easier. Moreover, appropriate counselling, reassurance and response to side-effects are key for continuation of hormonal method. Counselling regarding managing side-effects by lady health workers at home and by health care providers at centers should be ensured. In future, a cohort study to observe behavior of women for using, continuing, switching over and discontinuing hormonal method would add to knowledge regarding continuous use of hormonal methods.

Dissemination Plan

The findings of this study will be presented at important national and international conferences and published in peer-reviewed scientific journals.

Ethics approval and consent to participate

This study was approved by Ethical Review Committee (ERC) Aga Khan University (2022-6023-21033). All methods were performed in accordance with the ethical guidelines and regulations of ERC Aga Khan University. Informed consent was taken from each respondent before data collection of the study. Participants were assured of confidentiality and privacy.

Consent of Publication

Not applicable

Availability of data and materials

The dataset used during the current study is available from the corresponding author on reasonable request.

Competing Interests

The authors have declared that no competing interests exist.

Patient and public involvement

Patients and / or the public were not involved in the design, conduct, or reporting, or dissemination plans of this research.

For peer review only - http://bmjopen.bmj.com/site/about/guidelines.xhtml

For peer review only

Funding

Funding from HRP Alliance, part of UNDP-UNFPA-UNICEF-WHO-World Bank special program of research, development, and Research Training in Human Reproduction (HRP), a cosponsored program executed by The World Health Organization (WHO). The main funders HRP had no role in study design, data collection and analysis.

Grant Number

Not Applicable

Authors contribution

Fazila Bibi

Roles: Conceptualization, Data Curation, Data Analysis, investigation, Methodology, Software, Original draft, writing.

Affiliation: Center of Excellence for Trauma and Emergencies, Aga Khan University, Karachi, Pakistan

Sarah Saleem

Roles: Conceptualization, Methodology, Data Curation, Supervision, Writing-Review and Editing.

Affiliation: Department of Community Health Sciences, Aga Khan University, Karachi, Pakistan

Shiyam Sunder

Roles: Methodology, Writing-Review and Editing

Affiliation: Department of Community Health Sciences, Aga Khan University, Karachi, Pakistan

Shafquat Rozi

Roles: Data Analysis, Editing

For peer review only - http://bmjopen.bmj.com/site/about/guidelines.xhtml

Affiliation: Department of Community Health Sciences, Aga Khan University, Karachi, Pakistan

Acknowledgements

Fazila Bibi received funding from HRP Alliance, part of UNDP-UNFPA-UNICEF-WHO-World Bank special program of research, development, and Research Training in Human Reproduction (HRP), a cosponsored program executed by The World Health Organization (WHO) to complete her studies. This article represents the views of the named authors only and does not represent the views of World Health Organization.

References

1. Mustafa G, Azmat SK, Hameed W, Ali S, Ishaque M, Hussain W, et al. Family Planning Knowledge, Attitudes, and Practices among Married Men and Women in Rural Areas of Pakistan: Findings from a Qualitative Need Assessment Study. Int J Reprod Med. 2015; 2015:190520.

2. Ontiri S, Mutea L, Naanyu V, Kabue M, Biesma R, Stekelenburg J. A qualitative exploration of contraceptive use and discontinuation among women with an unmet need for modern contraception in Kenya. Reprod Health. 2021;18(1):33.

3. Affairs UNDoEaS. Contraceptive use by method. 2019. https://www.un.org/development/desa/pd/sites/www.un.org.development.desa.pd/files/files/documents/2020/Jan/un_2019_contraceptiveusebymethod_databooklet.pdf

4. Thobani R, Jessani S, Azam I, Reza S, Sami N, Rozi S, et al. Factors associated with the discontinuation of modern methods of contraception in the low-income areas of Sukh Initiative Karachi: A community-based case control study. PLoS One. 2019;14(7):e0218952.

5. Bank W. Family planning in South Asia: Improving equity of access [Internet]2012. [cited 2022]. Available from: https://blogs.worldbank.org/health/family-planning-in-south-asia-improving-equity-of-access.

For peer review only - http://bmjopen.bmj.com/site/about/guidelines.xhtml

6. Pakistan U. Multiple Indicator Cluster Survey 2016-17 Report and Key Findings - Gilgit Baltistan. 2017.

7. Okigbo CC, Speizer IS, Domino ME, Curtis SL. A Multilevel Logit Estimation of Factors Associated with Modern Contraception in Urban Nigeria. World Med Health Policy. 2017;9(1):65-88.

8. Todd N, Black A. Contraception for Adolescents. J Clin Res Pediatr Endocrinol. 2020;12(Suppl 1):28-40.

9. Hong R, Montana L, Mishra V. Family planning services quality as a determinant of use of IUD in Egypt. BMC Health Serv Res. 2006; 6:79.

10. Kungu W, Agwanda A, Khasakhala A. Prevalence of and factors associated with contraceptive discontinuation in Kenya. Afr J Prim Health Care Fam Med. 2022;14(1):e1-e11.

11. Sadat-Hashemi SM, Ghorbani R, Majdabadi HA, Farahani FK. Factors associated with contraceptive use in Tehran, Iran. Eur J Contracept Reprod Health Care. 2007;12(2):148-53.

12. Hall KS, Castaño PM, Westhoff CL. The influence of oral contraceptive knowledge on oral contraceptive continuation among young women. J Womens Health (Larchmt). 2014;23(7):596-601.

13. UNFPA. Mapping of Public and Private Sector for Family Planning and Reproductive Health Services in Selected Districts in AJK and GB - Report. 2020.

14. Shiferaw, S., Spigt, M., Seme, A., Amogne, A., Skrøvseth, S., Desta, S., Radloff, S., Tsui, A. and GeertJan, D., 2017. Does proximity of women to facilities with better choice of contraceptives affect their contraceptive utilization in rural Ethiopia. *PLOS ONE*, 12(11), p.e0187311.

15. Okwaraji YB, Webb EL, Edmond KM. Barriers in physical access to maternal health services in rural Ethiopia. BMC Health Serv Res. 2015; 15:493.

16. Aparna Sundaram RH, Zeba Sathar, Sabahat Hussain, Weissman EPaE. Adding It Up: Costs and Benefits of Meeting the Contraceptive and Maternal and Newborn Health Needs of Women in Pakistan. Population Council. 2019

17. Sumera Aziz Ali SS, Neelofar Sami, Mir Shabbar, Muhammad Ahmed. Geographic Access to Working Family Planning Centers and Unintended Pregnancies among Married Women: A Community Based Nested Case Control Study. Open Journal of Epidemiology. 2016.

18. Sumera Aziz Ali ZF. Predictors of modern contraceptive usage among women in rural Pakistan physical access to services still a major barrier. EC Gynecology. 2017.

For peer review only - http://bmjopen.bmj.com/site/about/guidelines.xhtml

19. Digitale J, Psaki S, Soler-Hampejsek E, Mensch BS. Correlates of Contraceptive Use and Health Facility Choice among Young Women in Malawi. Ann Am Acad Pol Soc Sci. 2017;669(1):93-124.

20. Aviisah PA, Dery S, Atsu BK, Yawson A, Alotaibi RM, Rezk HR, et al. Modern contraceptive use among women of reproductive age in Ghana: analysis of the 2003-2014 Ghana Demographic and Health Surveys. BMC Womens Health. 2018;18(1):141.

21. Rios-Zertuche D, Blanco LC, Zúñiga-Brenes P, Palmisano EB, Colombara DV, Mokdad AH, et al. Contraceptive knowledge and use among women living in the poorest areas of five Mesoamerican countries. Contraception. 2017;95(6):549-57.

22. Basu, A. M. (2005). Ultramodern contraception. *Asian Population Studies*, *1*(3), 303–323. doi:10.1080/17441730500441178

23. Hossain S, Sripad P, Zieman B, Roy S, Kennedy S, Hossain I, et al. Measuring quality of care at the community level using the contraceptive method information index plus and client reported experience metrics in Bangladesh. J Glob Health. 2021; 11:07007.

24. Culwell K, Hillard, P, Glob. Patient education and contraceptive compliance. 2008

25. Nisar S, Khan M, Nisar U. Impact Of Lady Health Workers On The Contraceptive Prevalence Rate In District Mardan. J Ayub Med Coll Abbottabad. 2020;32(1):104-10.

26. Tibaijuka L, Odongo R, Welikhe E, Mukisa W, Kugonza L, Busingye I, et al. Factors influencing use of long-acting versus short-acting contraceptive methods among reproductive-age women in a resource-limited setting. BMC Womens Health. 2017;17(1):25.

27. Westhoff CL, Heartwell S, Edwards S, Zieman M, Stuart G, Cwiak C, et al. Oral contraceptive discontinuation: do side effects matter? Am J Obstet Gynecol. 2007;196(4): 412.e1-6; discussion. e6-7.

For peer review only

For peer review only - http://bmjopen.bmj.com/site/about/guidelines.xhtml

For peer review only

For peer review only - http://bmjopen.bmj.com/site/about/guidelines.xhtml

Figure1: Map of Gilgit Baltistan showing distance in Kilometers from union council to family planning centers and distribution of LHWs in public and private family planning centers.

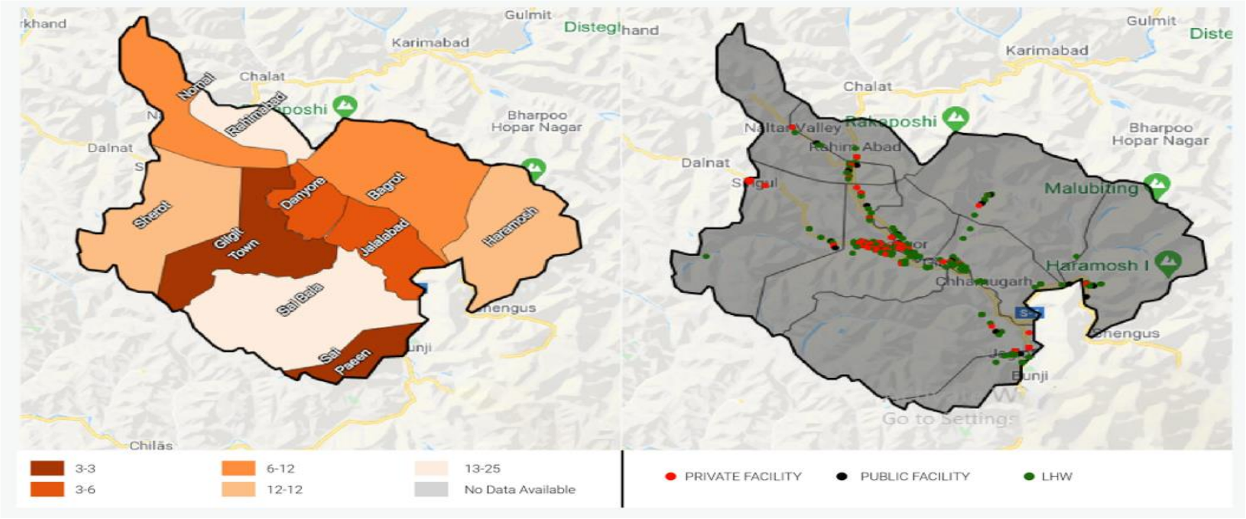

peer review only

STROBE Statement—Checklist of items that should be included in reports of ***case-control studies***

	Item No	Recommendation	Page No
**Title and abstract**	1	Indicate the study's design with a commonly used term in the title or the abstract<br>Case Control Study	
		(*b*) Provide in the abstract an informative and balanced summary of what was done and what was found<br>This study aimed to determine the factors associated with continuation of hormonal contraceptive methods among married women of Gilgit, Pakistan at least six months after their initiation. This was an Unmatched case control study conducted in the Community settings of district Gilgit, Pakistan from April 1, 2021, to July 30, 2021.The cases were married women of reproductive age who at the time of interview were using a hormonal method of contraception for at least last six months continuously, and controls were married women of reproductive age who had used hormonal method in the past and currently were using a non-hormonal method for at least last six months. Primary and Secondary outcome measures was OR for continuation of hormonal contraceptive.<br><br>The factors significantly associated with continuous use of hormonal contraceptive methods for our sample from Gilgit were family planning center at the walking distance from home (AOR 6.33, 95% CI, 3.74-10.71), satisfaction with current method in use (AOR 3.64 95% CI, 2.06-6.44), visits to the family planning center to avail services (AOR 1.86 95% CI, 1.07-3.45) and relatively older age of women (AOR 1.07, 95% C1, 1.02-1.12). In addition, women with formal education (AOR 0.27 95% CI, 0.12-0.6), were less likely to use a modern contraceptive method.<br><br>Continuation of a hormonal method use was associated with easy access to family planning centers, satisfaction with the current method use and frequent visits to the family planning centers. Continuation of hormonal method use was also seen in women with low education status. The importance of the presence of family planning centers near residential areas cannot be emphasized more. This not only provides easy access to FP methods, but also reassures women for continuation of modern methods when they face any unpleasant effects while using these.	
**Introduction**			
Background/rationale	2	Explain the scientific background and rationale for the investigation being reported.<br>Factors associated with contraceptive continuation are both methods related such as efficacy of the method, frequency of doses, and client- related such as age, education level, parity etc. Distance of the family planning center from home and availability of different methods at the center are additional important factors contributing to continuity in use of a method. The presence of healthcare staff at the center, level of education, age, number of living children and area of residence were all found to be associated with contraceptive continuation. Information regarding predictors of continuation of hormonal contraceptives is scarce from Pakistan and information related to continuation of any contraceptive method is mostly unavailable in Pakistan. Findings from this study would help in improving contraceptive prevalence rate and decreasing discontinuation of hormonal methods which are more effective compared to non-hormonal method. Moreover, promoting factors that are associated with the continuation of hormonal method will help to avoid	

For peer review only - http://bmjopen.bmj.com/site/about/guidelines.xhtml

		untimed and unwanted pregnancies hence it will improve maternal health.	
Objectives	3	State specific objectives, including any prespecified hypotheses.<br>This study aimed to determine the factors associated with continuation of hormonal contraceptive methods among married women of Gilgit, Pakistan at least six months after their initiation.<br>**Hypothesis**<br>The women who use hormonal methods continuously for six months are two times more at Odds of living at less than 30 minutes walking distance from a FP center as compared to those who discontinue to use a method within six months of starting it.	
**Methods**			
Study design	4	Present key elements of study design early in the paper<br>We use a community-based unmatched case control study design, with ratio of cases to control as 1:2.	
Setting	5	Describe the setting, locations, and relevant dates, including periods of recruitment, exposure, follow-up, and data collection.<br>We collected data from the catchment population of LHWs of 3 union councils Gilgit town, Danyore, and Nomal. Data were collected from March 2021 to July 2021 by lady health visitors who were trained for data collection.	
Participants	6	Give the eligibility criteria, and the sources and methods of case ascertainment and control selection. Give the rationale for the choice of cases and controls<br>Hormonal method users were sexually active women who were using one of the hormonal contraceptive methods i.e., pills, injectables or implants for at least last six months without disruption at the time of enrolment in the study and Controls were sexually active married women of reproductive age, with history of using a hormonal method but stopped it before six months after initiating it and switched to a non-hormonal contraceptive method for at least last six months. We excluded women using intrauterine contraceptive device (IUCD), as there are of two types (hormonal and non-hormonal) methods and women might not know the type of IUCD she was using. Women who were using permanent method of contraception were also excluded	
		(*b*) For matched studies, give matching criteria and the number of controls per case	
Variables	7	Clearly define all outcomes, exposures, predictors, potential confounders, and effect modifiers. Give diagnostic criteria, if applicable<br>Contraceptive continuation is the process of uninterrupted usage of contraceptive methods for at least six months.	
Data sources/ measurement	8*	For each variable of interest, give sources of data and details of methods of assessment (measurement). Describe comparability of assessment methods if there is more than one group<br>The study Questionnaire was designed after reviewing literature, PDHS 2017-18 questionnaire and service provision assessment survey form of Measure. The Questionnaire was translated into Urdu to make it understandable for study participants. We used Urdu as our medium of collecting information because it is our national language which is understood and spoken by a large majority (66.9 %) of young people in in GB (MICS 2017).	

For peer review only - http://bmjopen.bmj.com/site/about/guidelines.xhtml

For peer review only

Bias	9	Describe any efforts to address potential sources of bias	
Study size	10	Explain how the study size was arrived at<br>**Sample size estimation**<br>We used open epi software to estimate sample size of the study. The prevalence of various factors [(distance (32.12%), counselling provided by healthcare providers (62.77%), and experienced side effects (80.29)] for controls and cases were obtained from the literature search. We calculated sample size for all three factors mentioned and have selected range that is covering all three factors. To account for non-response rate we have increased sample size by 20% so, to determine an odds ratio of at least 2, by taking power of 80 percent and at significance of 5 percent with the ratio of 1:2 between cases and controls, a minimum of 141 cases and 282 controls were required for this study using 32.12 percent exposure in controls from literature.	
Quantitative variables	11	Explain how quantitative variables were handled in the analyses. If applicable, describe which groupings were chosen and why<br>Descriptive statistics were calculated for both categorical and continuous variables. Frequency and percentages have been reported for qualitative variables such as area of residence, level of education, socioeconomic status, cost of services, mode of transport, distance from home to FP center, decision maker of contraceptive use, source of current method, information given by LHW, satisfaction with home services, FP counselling and information related to side effects experienced by women. Mean and standard deviation is reported for quantitative variables such as age of woman, age at first contraceptive use, number of living children and number of FP center operational days.	
Statistical methods	12	Describe all statistical methods, including those used to control for confounding<br>Binary logistic regression	
		(*b*) Describe any methods used to examine subgroups and interactions	
		(*c*) Explain how missing data were addressed<br>there were no significant missing values	
		(*d*) If applicable, explain how matching of cases and controls was addressed	
		(*e*) Describe any sensitivity analyses	
**Results**			
Participants	13*	Report numbers of individuals at each stage of study—eg numbers potentially eligible, examined for eligibility, confirmed eligible, included in the study, completing follow-up, and analysed<br>number of potential eligible: 520<br>examined for eligibility: 499<br>confirm eligible:423	
		(b) Give reasons for non-participation at each stage	
		(c) Consider use of a flow diagram	
Descriptive data	14*	Give characteristics of study participants (eg demographic, clinical, social) and information on exposures and potential confounders<br>For cases, we enrolled 141 women who were using one of the hormonal	

For peer review only - http://bmjopen.bmj.com/site/about/guidelines.xhtml

		methods (Pills, injections and Implants) for last six months without disruption and 282 women who ever used hormonal method in past and currently were using non-hormonal method from last six months (discontinued users). Among cases, 24.1 percent of the women were using pills, 2.1 percent of the women were using implant and 73.4 percent were using injectables for contraception. Traditional method was more common among controls. 59.2 percent of the women were using traditional methods and 40.7 percent of the women were using condoms. Previously, 62 percent of the women among controls discontinued pills, 0.35 percent discontinued implant and 37.5 percent of the women discontinued injectables and started to use non-hormonal method.	
		(b) Indicate number of participants with missing data for each variable of interest	
Outcome data	15*	Report numbers in each exposure category, or summary measures of exposure	

For peer review only

For peer review only - http://bmjopen.bmj.com/site/about/guidelines.xhtml

Main results	16	(*a*) Give unadjusted estimates and, if applicable, confounder-adjusted estimates and their precision (eg, 95% confidence interval). Make clear which confounders were adjusted for and why they were included	
		(*b*) Report category boundaries when continuous variables were categorized	
		(*c*) If relevant, consider translating estimates of relative risk into absolute risk for a meaningful time period	
Other analyses	17	Report other analyses done—eg analyses of subgroups and interactions, and sensitivity analyses	
**Discussion**			
Key results	18	Summarise key results with reference to study objectives<br>the model we concluded that odds of a walking distance of at most 30 minutes from home to a family planning center is 6.33 times more among continued users compared to discontinued users (95% CI=3.74-10.71). For every one-year increase in age of the women among continued users, the increase in log odds of hormonal contraceptive continuation was 1.07 times compared to discontinued users (adjusted OR=1.07, CI=1.02-1.12). Odds of having formal education was 73% less among continued users compared to discontinued users (adjusted OR=0.27, 95% CI=0.07-0.89). Odds of being satisfied with current method was 3.64 times more among continued users compared to discontinued users (95% CI=2.06-6.44). Odds of visiting to family planning center was 1.86 times more among continued users compared to discontinued users (95% CI=1.07-3.45).	
Limitations	19	Discuss limitations of the study, taking into account sources of potential bias or imprecision. Discuss both direction and magnitude of any potential bias<br>Major limitation of the study was Subjective assessment of Primary exposure (taken on face value) due to unavailability of internet access in all union councils of district Gilgit. Women might have given wrong responses. Furthermore, this study is generalizable to areas that are geographically similar to district Gilgit.	
Interpretation	20	Give a cautious overall interpretation of results considering objectives, limitations, multiplicity of analyses, results from similar studies, and other relevant evidence<br>In Gilgit, continuation of hormonal method use was associated with easy access to family planning centers, satisfaction with current method use and visits to FP center. In addition, continuation was also seen in women with no formal education. Women who were using hormonal method continuously were three years older than discontinued users. Despite experiencing side-effects women who were using hormonal contraceptives continued to use method of their choice for at least six months.	
Generalisability	21	Discuss the generalisability (external validity) of the study results<br>this study is generalizable to areas that are geographically similar to district Gilgit.	
**Other information**			
Funding	22	Give the source of funding and the role of the funders for the present study and, if applicable, for the original study on which the present article is based<br>Funding from HRP Alliance, part of UNDP-UNFPA-UNICEF-WHO-World Bank special program of research, development, and Research Training in Human Reproduction (HRP), a cosponsored program executed by The World Health Organization (WHO). The main funders HRP had no role in study design, data collection and analysis.	

*Give information separately for cases and controls.

For peer review only - http://bmjopen.bmj.com/site/about/guidelines.xhtml

Note: An Explanation and Elaboration article discusses each checklist item and gives methodological background and published examples of transparent reporting. The STROBE checklist is best used in conjunction with this article (freely available on the Web sites of PLoS Medicine at http://www.plosmedicine.org/, Annals of Internal Medicine at http://www.annals.org/, and Epidemiology at http://www.epidem.com/). Information on the STROBE Initiative is available at http://www.strobe-statement.org.

For peer review only

For peer review only - http://bmjopen.bmj.com/site/about/guidelines.xhtml

BMJ Open

Factors associated with continuation of hormonal contraceptives among married women of reproductive age in Gilgit- Pakistan: a community-based case control study

Journal:	*BMJ Open*
Manuscript ID	bmjopen-2023-075490.R2
Article Type:	Original research
Date Submitted by the Author:	04-Oct-2023
Complete List of Authors:	Bibi, Fazila; The Aga Khan University, Center of Excellence for Trauma and Emergencies<br>Saleem, Sarah; Aga Khan University, Community Health Sciences<br>Tikmani, Shiyam; Aga Khan University, Community Health Sciences<br>Rozi, Shafquat; Aga Khan University, Community Health Sciences
**Primary Subject Heading**:	Obstetrics and gynaecology
Secondary Subject Heading:	Sexual health, Public health
Keywords:	SEXUAL MEDICINE, REPRODUCTIVE MEDICINE, GYNAECOLOGY, Health Services Accessibility, Reproductive medicine < GYNAECOLOGY

SCHOLARONE™ Manuscripts

I, the Submitting Author has the right to grant and does grant on behalf of all authors of the Work (as defined in the below author licence), an exclusive licence and/or a non-exclusive licence for contributions from authors who are: i) UK Crown employees; ii) where BMJ has agreed a CC-BY licence shall apply, and/or iii) in accordance with the terms applicable for US Federal Government officers or employees acting as part of their official duties; on a worldwide, perpetual, irrevocable, royalty-free basis to BMJ Publishing Group Ltd ("BMJ") its licensees and where the relevant Journal is co-owned by BMJ to the co-owners of the Journal, to publish the Work in this journal and any other BMJ products and to exploit all rights, as set out in our licence.

The Submitting Author accepts and understands that any supply made under these terms is made by BMJ to the Submitting Author unless you are acting as an employee on behalf of your employer or a postgraduate student of an affiliated institution which is paying any applicable article publishing charge ("APC") for Open Access articles. Where the Submitting Author wishes to make the Work available on an Open Access basis (and intends to pay the relevant APC), the terms of reuse of such Open Access shall be governed by a Creative Commons licence – details of these licences and which Creative Commons licence will apply to this Work are set out in our licence referred to above.

Other than as permitted in any relevant BMJ Author's Self Archiving Policies, I confirm this Work has not been accepted for publication elsewhere, is not being considered for publication elsewhere and does not duplicate material already published. I confirm all authors consent to publication of this Work and authorise the granting of this licence.

For peer review only

Factors associated with continuation of hormonal contraceptives among married women of reproductive age in Gilgit- Pakistan: a community-based case control study.

Fazila Bibi[1*], Sarah Saleem[2], Shiyam Sunder[3], Shafquat Rozi[4]

[1]Center of Excellence for Trauma and Emergencies, Aga Khan University, Karachi, Pakistan

[2]Community Health Sciences, Aga Khan University, Karachi, Pakistan

[3]Community Health Sciences, Aga Khan University, Karachi, Pakistan

[4]Community Health Sciences, Aga Khan University, Karachi, Pakistan

Author information:

Fazila Bibi (FB)- Fazila.sahibjan@aku.edu

Sarah Saleem (SS)- sarah.saleem@aku.edu

Shiyam Sunder (SS) Shiyam.sunder@aku.edu

Shafquat Rozi (SR)- Shafquat.rozi@aku.edu

Correspondence to: Fazila.sahibjan@aku.edu

Centre of Excellence for Trauma and Emergencies, Aga Khan University, Karachi,

Address: Stadium Road, P.O. Box-3500, Karachi (74800), Pakistan.

Tel: 92-21-3486-4837

Fax: 92-21-34932095

For peer review only - http://bmjopen.bmj.com/site/about/guidelines.xhtml

ABSTRACT

Objective

This study aimed to determine the factors associated with continuation of hormonal contraceptive methods among married women of Gilgit, Pakistan at least six months after their initiation.

Design

Unmatched case control study.

Setting

Community settings of district Gilgit, Pakistan from April 1, 2021, to July 30, 2021.

Participants

The cases were married women of reproductive age who at the time of interview were using a hormonal method of contraception for at least last six months continuously, and controls were married women of reproductive age who had used hormonal method in the past and currently were using a non-hormonal method for at least last six months.

Primary and Secondary outcome measures

OR for continuation of hormonal contraceptive.

Results

The factors significantly associated with continuous use of hormonal contraceptive methods for our sample from Gilgit were family planning center at the walking distance from home (AOR 6.33, 95% CI, 3.74-10.71), satisfaction with current method in use (AOR 3.64 95% CI, 2.06-6.44), visits to the family planning center to avail services (AOR 1.86 95% CI, 1.07-3.45) and relatively older age of women (AOR 1.07, 95% C1, 1.02-1.12). In addition, women with formal education (AOR 0.27 95% CI, 0.12-0.6), were less likely to use a modern contraceptive method.

Conclusion

For peer review only - http://bmjopen.bmj.com/site/about/guidelines.xhtml

For peer review only

Continuation of a hormonal method use was associated with easy access to family planning centers, satisfaction with the current method use and frequent visits to the family planning centers. Continuation of hormonal method use was also seen in women with low education status. The importance of the presence of family planning centers near residential areas cannot be emphasized more. This not only provides easy access to FP methods, but also reassures women for continuation of modern methods when they face any unpleasant effects while using these.

Strengths and limitations of this study

- Case to control ratio was kept 1:2 which enhances the likelihood that our findings are representative of the experiences of the general population likely to represent experience of general population.
- Subjective assessment of walking distance due to lack of internet access in all union councils of District Gilgit.
- This study is generalizable to areas that are geographically similar to district Gilgit.
- Data was collection was done during Covid19 times; women might have discontinued going to family planning centers for hormonal contraceptives due to fear of getting infection and this might have affected our results.

For peer review only - http://bmjopen.bmj.com/site/about/guidelines.xhtml

Introduction

Access to safe and effective contraceptives is the right of every woman and man regardless of their religion, or socio-economic status, region, or country. This ensures that individuals can make well-informed decisions about the contraceptive method that best suit their preferences and needs (1). The trends of contraceptive use, its discontinuation and switching a method are significant identifiers of how well family planning programs are fulfilling the needs of family planning of reproductive age women and couples in a country (2). Worldwide 922 million women of reproductive age use any form of contraceptives (3), however, there are still 12 percent of the women who want to avoid pregnancy and are not using any method of contraception (4). High fertility rates and low contraceptive prevalence (CPR) rates are the main elements of exponential population growth in South Asian countries (5). In Pakistan, the combination of a high fertility rate and low contraceptive utilization plays a significant role in contributing to unfavorable reproductive health indicators, particularly concerning maternal mortality. Furthermore, discontinuation of contraception has been found to be one of the major reasons of induced abortions in Pakistan. Multi-indicator cluster survey of Pakistan (MICS 2016-17) reported that in Gilgit Baltistan (GB) 38.1% of married women of reproductive age had access to contraception, of these 32.1% use one of the modern contraceptive methods (6).

Contraceptive continuation is the process of uninterrupted usage of contraceptive methods for at least six months. Hormonal methods use hormones to regulate ovulation and prevent pregnancy that include injectable birth controls, contraceptive skin patch, vaginal ring and oral contraceptive pills. Factors associated with contraceptive continuation are both methods related such as efficacy of the method, frequency of doses, and client- related such as age, education level, parity etc. (7,8). Distance of the family planning center from home and

For peer review only - http://bmjopen.bmj.com/site/about/guidelines.xhtml

For peer review only

availability of different methods at the center are additional important factors contributing to continuity in use of a method. Presence of healthcare staff at the center, level of education, age, number of living children and area of residence were all found to be associated with contraceptive continuation (9,10,11,12).

There is notable scarcity of information regarding predictors of sustained use of hormonal contraceptives in Pakistan, and information concerning the continuation of any contraceptive method is generally lacking. Findings from this study could play a pivotal role in enhancing contraceptive prevalence rate and decreasing discontinuation of hormonal methods, which are more effective when compared to non-hormonal method alternatives. Additionally, promoting factors linked to the continued use of hormonal method can aid in preventing unintended pregnancies, thereby contributing to improved maternal health outcomes.

Gilgit Baltistan is situated in Northern part of Pakistan which covers an area of over 72,971 KM^2 and is highly hilly and mountainous. Gilgit is the capital and largest district with estimated population of 216,760 (13). Seventy-nine percent of family planning centers with trained staff in Gilgit are in the main town while remaining are in other union councils with 4-29 % trained staff (13) as shown in figure1.

This suggests that all specialized facilities are centered in Gilgit town and women from other union councils must travel to get specialized services and the average distance from Gilgit town to the nearest health facility is 2.7 kilometers which is far less than other union councils that are 18-24 kilometers away from the nearest health facility (13). Considering the high efficacy of the hormonal methods to prevent pregnancy, we were interested in identifying region-specific (Gilgit) factors that were associated with the continuation of hormonal contraceptives.

Methods

For peer review only - http://bmjopen.bmj.com/site/about/guidelines.xhtml

We used a community-based unmatched case control study design, with ratio of cases to control as 1:2. Cases were married women of reproductive age who were currently using a hormonal method of contraception for at least last six months, and controls were married women of reproductive age who had ever used a hormonal method but now are using a non-hormonal method at least for last six months, This was to know why women switched from hormonal to non-hormonal method and to make groups comparable we selected control group with history of hormonal method use. We collected data from the catchment population of LHWs of 3 union councils Gilgit town, Danyore, and Nomal. Hormonal method users were sexually active women who were using one of the hormonal contraceptive methods i.e., pills, injectables or implants for at least last six months without disruption at the time of enrolment in the study and Controls were sexually active married women of reproductive age, with history of using a hormonal method but stopped it before six months after initiating it and switched to a non-hormonal contraceptive method for at least last six months. We excluded women using intrauterine contraceptive device (IUCD), as there are of two types (hormonal and non-hormonal) methods and women might not know the type of IUCD she was using. Women who were using permanent method of contraception were also excluded. Data were collected from March 2021 to July 2021. Written informed consent was obtained from all eligible women who agreed to participate in the study. This study was approved by ethical review committee of Aga Khan University.

To account for non-response rate, sample size was increased by 20% so, to determine an odds ratio of at least 2, by taking power of 80% and at significance of 5% with the ratio of 1:2 between cases and controls, a minimum of 141 cases and 282 controls were required for this study (4).

Data were analyzed using Stata version 14. Frequency and percentages are reported for qualitative variables. Mean and standard deviation are reported for continuous quantitative

For peer review only - http://bmjopen.bmj.com/site/about/guidelines.xhtml

variables. We used logistic regression for both univariate (simple logistic regression) and multivariable analysis to identify factors associated with continued use of a hormonal contraceptive method. Multicollinearity among all independent variables significant at univariate level was checked considering 0.80 as cut off for Pearson correlation (for continuous variables), Crammers V to check multicollinearity among categorical variables.

Patient and Public Involvement

No patient and public involvement in current study.

Results

Description of study population

Table1

Socio- demographic characteristics of continued and discontinued users of hormonal contraceptives.

Variables	Continued users n=141 n (%)	Discontinued users n=282 n (%)
**Age of women (years)**		
**Mean (SD)**	34.8 (5.9)	31.5 (5.3)
**Area of residence**		
**Rural**	45 (31.9)	64 (22.7)
**Urban**	96 (68.0)	218 (77.3)
**Education**		
**No formal education**	30 (21.2)	18 (6.3)

For peer review only - http://bmjopen.bmj.com/site/about/guidelines.xhtml

**Formal education**	111 (78.7)	264 (93.6)
**Language**		
**Brushaski**	96 (68.0)	204 (72.3)
**Others**	45 (31.9)	78 (27.6)

We completed interviews on 141 cases and 282 controls. Table 1 illustrates socio-demographic characteristics of continued users of hormonal method for at least last six months and current non-hormonal method users who used hormonal method and discontinued users. The mean age of women who were using hormonal method was 34.8 ± 5.9 years whereas mean age for discontinued user was 31.5 ± 5.3 years.

Table 2

Frequency distribution of biological factors among continued users and discontinued users of hormonal contraceptives.

**Variables**	**Continued users n=141 n (%)**	**Discontinued users n=282 n (%)**
**Age at initiating contraceptive use**		
**Mean (SD)**	26.2 (3.8)	25.4 (3.1)
**Number of living children at the time of initiating contraceptive use**		
**Mean (SD)**	2 (1.4)	1 (1.2)

For peer review only - http://bmjopen.bmj.com/site/about/guidelines.xhtml

**Decision maker of contraceptive use**		
**Self**	18 (12.7)	22 (7.8)
**Mainly Husband**	33 (23.4)	136 (48.2)
**Joint decision**	90 (63.8)	124 (43.9)
**Source of current method**		
**Government source**	114 (81.4)	90 (78.9)
**Private source**	26 (18.5)	24 (21.0)
**Information given regarding side effects**		
**Yes**	124 (87.9)	260 (87.9)
**No**	17 (12.0)	22 (7.8)
**Information given regarding other methods**		
**Yes**	**98 (69.5)**	**214 (75.8)**
**No**	**43 (30.5)**	**68 (24.1)**
**Experienced side effects**		
**Yes**	119 (84.4)	7 (2.4)
**No**	22 (15.6)	275 (97.5)
**Satisfied with current method**		
**Yes**	118 (83.6)	168 (59.5)
**No**	23 (16.3)	114 (40.4)

For peer review only - http://bmjopen.bmj.com/site/about/guidelines.xhtml

**Want more effective method**		
**Yes**	125 (44.3)	35 (24.8)
**No**	157 (55.6)	106 (75.1)
**Information given by LHW**		
**Mother and child health**	6 (4.2)	65 (23.0)
**Contraceptive supplies, Family planning and family health counseling**	120 (85.1)	209 (74.1)
**Others**	15 (10.6)	8 (2.8)

Table 2 presents the frequency distribution of biological factors among women who continued using hormonal contraceptives and those who discontinued their use. Among continued users, at the time of interview, 24.1% of the women were using pills, 2.1% of the women were using implant and 73.4% were using injectables for contraception. Conversely, among discontinued users, nearly 59.0%of the women were using traditional methods and 40.7% of the women were using condoms at the time of the interview. Before transitioning to non-hormonal methods these women were using pills (62.0%), injectables (37.5%) and implants (0.35%). Mean age at initiating contraceptive use among continued users was 26.2 years (SD 3.8) and among discontinued users 25.4 years (SD 3.1 years). Notably, there were some differences observed between the two groups in terms of number of living children they had at the time of initiating contraceptive use. Continued users had a mean of 2 children (SD 1.4 years) while discontinued users had one child (SD 1.2 years).

Factors related to service delivery.

Table 3

Frequency distribution of factors related to service delivery among continued and discontinued users.

Variables	Continued users n=141	Discontinued users n=282

For peer review only - http://bmjopen.bmj.com/site/about/guidelines.xhtml

	n (%)	n (%)
**Ever been to FP centers to avail services**		
**Yes**	119 (84.4)	205 (72.7)
**No**	22 (15.6)	77 (27.3)
**Reasons for not going to FP centers**		
**Facility related issues**	4 (18.1)	27 (35.0)
**Services related issues**	13 (59.0)	34 (44.1)
**Transport related issues**	5 (22.7)	16 (20.7)
**Number of days services provided by center**		
**Mean (SD)**	6.0 (1)	5.7 (0.9)
**Routine fee charged by facility**		
**Yes**	133 (94.3)	277 (98.2)
**No**	8 (5.6)	5 (1.7)
**Presence of healthcare staff at center**		
**Yes**	105 (74.4)	163 (57.8)
**No**	36 (25.5)	119 (42.2)
**Counseling services at FP center**		
**Yes**	75 (53.1)	133 (47.1)
**No**	66 (46.8)	149 (52.8)
**Satisfied with services provided at home**		
**Yes**	92 (65.2)	218 (77.3)
**No**	49 (34.7)	64 (22.7)

For peer review only - http://bmjopen.bmj.com/site/about/guidelines.xhtml

For peer review only

**Reasons for dissatisfaction**		
**Did not like method**	12 (24.4)	10 (15.6)
**Other (have more side-effects, provide counselling only)**	37 (75.5)	54 (84.3)
**Distance from home to FP center**		
**Near to my house at walking distance**	111 (78.7)	108 (38.3)
**Far need transport**	30 (21.2)	174 (61.7)
**Cost of round trip in USD**		
**No cost**	110 (80.1)	112 (39.7)
**≤0.4**	5 (1.4)	55 (19.5)
**≤2**	17 (12.0)	78 (27.6)
**>2**	9 (6.3)	37 (13.1)

Table 3 provides an overview of factors related to service delivery among continued and discontinued users. All the women in our sample were aware of the nearest family planning centers of their area. Around eighty four percent (84.4%) of continued users and 73.0% of discontinued users had been to FP centers to avail services in the last six months. Walking distance from home to nearest family planning center was measured subjectively by asking women if they could walk to access nearest center on foot or require transport to reach to the center. Approximately, 79.0% of the continued users mentioned that the family planning center is within walking distance while 21.2% needed a transport to reach to the nearest

For peer review only - http://bmjopen.bmj.com/site/about/guidelines.xhtml

family planning center. In contrast thirty-eight (38.3%) % of the discontinued users were able to reach the center on foot while 61% needed transport.

In both the groups, those women who did not visit FP clinics mentioned unsuitable timings of the FP center, far distance, non-availability of FP staff, cost of services, unavailability of desired method and transportation issues as some of the reasons for not availing the services.

Women were asked about their satisfaction from the services provided at home by the LHWs. It was found that 65.2% of women among continued users and 77.3% women among discontinued users expressed satisfaction with the home services they received.

Table 4 Adjusted odds ratio (95% confidence interval) for the factors associated with continuation of hormonal contraceptives among continued and discontinued users in Gilgit, Pakistan.

Variables	Continued users n=141 n (%)	Discontinued users n=298 n (%)	adjusted odds ratio (95% C.I)
**Distance from home to FP center**			
**need transport**	30 (21.2)	174 (61.7)	1
**at walking distance**	111 (78.7)	108 (38.3)	6.4 (3.70-11.19)
**Age of women (years)**			
**Mean (SD)**	34.8 (5.9)	31.5 (5.3)	1.06 (1.01-1.11)
**Education**			

For peer review only - http://bmjopen.bmj.com/site/about/guidelines.xhtml

**No formal education**	30 (21.2)	18 (6.3)	1
**Formal Education**	111 (78.7)	264 (93.6)	0.27 (0.13-0.47)
**Satisfied with current method**			
**No**	23 (16.3)	114 (40.4)	1
**Yes**	118 (83.6)	168 (59.5)	3.64 (2.06-6.44)
**Visits FP center**			
**No**	22 (15.6)	77 (27.3)	1
**Yes**	119 (84.4)	205 (72.7)	1.86 (1.07-3.45)

*Only statistically significant variables are shown in table 4

On multivariable analysis after adjusting for effect of other variables in the model walking distance to a family planning center (AOR=6.33, 95% CI=3.74-10.71) visiting family planning center (AOR=1.86, 95% CI=1.07-3.45), older age of the women (AOR=1.07, CI=1.02-1.12), having formal education (AOR 0.27, 95% CI=0.13-0.47) were independently associated with continuation of hormonal contraceptives as shown in Table 4.

Discussion

The findings of our study highlight the importance of both service delivery and individual factors in influencing the continuation of hormonal contraceptive use for a minimum of six months among women in Gilgit Baltistan. Among the service delivery-related factors, significant contributors included the proximity of the family planning center to the women

For peer review only

residential area, the quality of information provided by the family planning center staff regarding method side effects, overall satisfaction with the current contraceptive method, and regular visits to the nearest 1family planning center. Additionally, noteworthy individual factors that played a significant role were age and education status of the women.

Our results are in concordance with other studies (14). Proximity to the health facilities and facilities providing wide range of contraceptives increases of the use of contraceptive methods, Physical access to family planning services and other maternal and neonatal health facilities is mostly determined by distance to travel. (15,16,17,18). Access to family planning centers within 1.9 km and that provide four or more methods increase contraceptive use and as distance increases from two kilometers irrespective of number of methods provided by the facility the contraceptive use starts to decline (19). Previous studies from Pakistan also found that proximity to functional family planning centers is strongly associated with contraceptive use (16,18). Our study also suggests that walking distance from home to family planning centers matters for the use of a contraceptive method.

In our study sample, women who had ever been to formal school were less likely to use a hormonal contraceptive compared to those who had never been to formal school. Conventionally, women with no formal education are less likely to practice modern contraceptives compared with some formal education (20) and women's education was strong predictor of contraceptive use and women who had attained secondary or more education were twice likely to avail contraceptive services (21). However, there can be religious reasons for preferring non-invasive and non-chemical methods. Upper class women face more facilitating conditions for traditional contraceptive use such as better husband wife communication, which is so important for effective birth control and more importantly, they may be better able to deal with contraceptive failure because they have capacity to afford an additional child and they also have easy access to abortion (22). We believe, in our study, closer distance and easy access

For peer review only - http://bmjopen.bmj.com/site/about/guidelines.xhtml

to contraceptive method of choice has made education less important as a factor for women to use of a contraceptive method. In context of Gilgit, living near to FP centers and door to door visits by LHWs has made women of the region more aware about the contraceptive methods irrespective of education status.

Our study found that satisfaction with the method in current use is one of the significant reasons to continue hormonal method of contraception. Client's satisfaction with a method makes users compliant to use and becomes a main factor for its continuation. In our study, all the women said that they have been visited by a lady health worker in last six months and they had received contraceptive counselling (side-effects, how to manage side-effects and seek treatment in case of experiencing side-effects) and referral. Women who have been visited by a lady health worker and discussed family planning in the last twelve months is positively associated with continuing long-acting reversible contraceptives (23), similarly, women in Malawi who had been visited by lady health workers were more likely to continue injectables compared to other contraceptives (19). Hence lady health workers are critical source of information for introducing women to hormonal contraceptives (24). LHW's in Gilgit district provide door to door contraceptive counselling and make women aware of possible side-effects of a contraceptive method and refer them and counsel them about ways to manage and seek treatment for the side-effects. We found positive association of contraceptive use with age. Women aged 20-44 prefer short acting compared to women aged 15-19 who prefer long-acting contraceptives (25,26,27). As family of a woman completes, she desires to limit pregnancies and hence contraceptive compliance increases as age of the women increases (25,26).

Our study possessed several notable strengths. Firstly, we employed a robust recruitment process based on lady health workers' records, who routinely update and maintain accurate records during their frequent visits. Additionally, we corroborated this information with the

For peer review only - http://bmjopen.bmj.com/site/about/guidelines.xhtml

women themselves before enrolling them in the study. This method significantly reduced the likelihood of misclassifying the outcome status of the participants.

Furthermore, we maintained a balanced case to control ratio 1:2, which enhances the likelihood that our findings are representative of the experiences of the general population. We also employed a consistent data collection tool for both cases and controls, ensuring the reliability and comparability of the data.

However, it is important to acknowledge the primary limitation of our study, which was the subjective assessment of walking distance. This limitation arose from the lack of internet access in all union councils of District Gilgit. Nevertheless, women who never used transport to reach FP is supportive of giving an affirmation of the proximity of FP centers from their homes. Additionally, our data collection occurred during COVID-19 pandemic, a period during which women may have refrained from visiting family planning centers due to concerns about contracting the virus. This factor could potentially have had an impact on our results. Furthermore, this study is generalizable to areas that are geographically similar district Gilgit.

Conclusion and recommendations

In Gilgit, continuation of hormonal method use was notably linked with easy access to family planning centers, satisfaction with current method use and visits to FP center. Our study findings recommends that family planning centers should be closer to the residential areas to make access of women easier. Moreover, appropriate counselling, reassurance and response to side-effects are key for continuation of hormonal method. Counselling regarding managing side-effects by lady health workers at home and by health care providers at centers should be ensured. Looking ahead, conducting a cohort study to observe behavior of women for using,

For peer review only - http://bmjopen.bmj.com/site/about/guidelines.xhtml

continuing, switching over and discontinuing hormonal method could provide valuable insights into the factors influencing the continuous use of these methods.

Dissemination Plan

The findings of this study will be presented at important national and international conferences and published in peer-reviewed scientific journals.

Ethics approval and consent to participate

This study was approved by Ethical Review Committee (ERC) Aga Khan University (2022-6023-21033). All methods were performed in accordance with the ethical guidelines and regulations of ERC Aga Khan University. Informed consent was taken from each respondent before data collection of the study. Participants were assured of confidentiality and privacy.

Consent of Publication

Not applicable

Availability of data and materials

The dataset used during the current study is available from the corresponding author on reasonable request.

Competing Interests

The authors have declared that no competing interests exist.

Patient and public involvement

Patients and / or the public were not involved in the design, conduct, or reporting, or dissemination plans of this research.

Funding

Funding from HRP Alliance, part of UNDP-UNFPA-UNICEF-WHO-World Bank special program of research, development, and Research Training in Human Reproduction (HRP), a

For peer review only - http://bmjopen.bmj.com/site/about/guidelines.xhtml

cosponsored program executed by The World Health Organization (WHO). The main funders HRP had no role in study design, data collection and analysis.

Grant Number

Not Applicable

Authors contribution

Fazila Bibi

Roles: Conceptualization, Data Curation, Data Analysis, investigation, Methodology, Software, Original draft, writing.

Affiliation: Center of Excellence for Trauma and Emergencies, Aga Khan University, Karachi, Pakistan

Sarah Saleem

Roles: Conceptualization, Methodology, Data Curation, Supervision, Writing-Review and Editing.

Affiliation: Department of Community Health Sciences, Aga Khan University, Karachi, Pakistan

Shiyam Sunder

Roles: Methodology, Writing-Review and Editing

Affiliation: Department of Community Health Sciences, Aga Khan University, Karachi, Pakistan

Shafquat Rozi

Roles: Data Analysis, Editing

Affiliation: Department of Community Health Sciences, Aga Khan University, Karachi, Pakistan

Acknowledgements

For peer review only - http://bmjopen.bmj.com/site/about/guidelines.xhtml

Fazila Bibi received funding from HRP Alliance, part of UNDP-UNFPA-UNICEF-WHO-World Bank special program of research, development, and Research Training in Human Reproduction (HRP), a cosponsored program executed by The World Health Organization (WHO) to complete her studies. This article represents the views of the named authors only and does not represent the views of World Health Organization.

Figure Legend/Caption map on right side: Average distance of public and private facilities in District Gilgit. Red dots show private facilities, black dots show public facilities and dark green shows lady health workers (LHW)

Figure legend/Caption left side: Map of District Gilgit

For peer review only

For peer review only - http://bmjopen.bmj.com/site/about/guidelines.xhtml

References

1. Mustafa G, Azmat SK, Hameed W, Ali S, Ishaque M, Hussain W, et al. Family Planning Knowledge, Attitudes, and Practices among Married Men and Women in Rural Areas of Pakistan: Findings from a Qualitative Need Assessment Study. Int J Reprod Med. 2015; 2015:190520.

2. Ontiri S, Mutea L, Naanyu V, Kabue M, Biesma R, Stekelenburg J. A qualitative exploration of contraceptive use and discontinuation among women with an unmet need for modern contraception in Kenya. Reprod Health. 2021;18(1):33.

3. Affairs UNDoEaS. Contraceptive use by method. 2019. https://www.un.org/development/desa/pd/sites/www.un.org.development.desa.pd/files/files/documents/2020/Jan/un_2019_contraceptiveusebymethod_databooklet.pdf

4. Thobani R, Jessani S, Azam I, Reza S, Sami N, Rozi S, et al. Factors associated with the discontinuation of modern methods of contraception in the low-income areas of Sukh Initiative Karachi: A community-based case control study. PLoS One. 2019;14(7):e0218952.

5. Bank W. Family planning in South Asia: Improving equity of access [Internet]2012. [cited 2022]. Available from: https://blogs.worldbank.org/health/family-planning-in-south-asia-improving-equity-of-access.

6. Pakistan U. Multiple Indicator Cluster Survey 2016-17 Report and Key Findings - Gilgit Baltistan. 2017.

7. Okigbo CC, Speizer IS, Domino ME, Curtis SL. A Multilevel Logit Estimation of Factors Associated with Modern Contraception in Urban Nigeria. World Med Health Policy. 2017;9(1):65-88.

8. Todd N, Black A. Contraception for Adolescents. J Clin Res Pediatr Endocrinol. 2020;12(Suppl 1):28-40.

9. Hong R, Montana L, Mishra V. Family planning services quality as a determinant of use of IUD in Egypt. BMC Health Serv Res. 2006; 6:79.

10. Kungu W, Agwanda A, Khasakhala A. Prevalence of and factors associated with contraceptive discontinuation in Kenya. Afr J Prim Health Care Fam Med. 2022;14(1):e1-e11.

11. Sadat-Hashemi SM, Ghorbani R, Majdabadi HA, Farahani FK. Factors associated with contraceptive use in Tehran, Iran. Eur J Contracept Reprod Health Care. 2007;12(2):148-53.

For peer review only - http://bmjopen.bmj.com/site/about/guidelines.xhtml

12. Hall KS, Castaño PM, Westhoff CL. The influence of oral contraceptive knowledge on oral contraceptive continuation among young women. J Womens Health (Larchmt). 2014;23(7):596-601.

13. UNFPA. Mapping of Public and Private Sector for Family Planning and Reproductive Health Services in Selected Districts in AJK and GB - Report. 2020.

14. Shiferaw, S., Spigt, M., Seme, A., Amogne, A., Skrøvseth, S., Desta, S., Radloff, S., Tsui, A. and GeertJan, D., 2017. Does proximity of women to facilities with better choice of contraceptives affect their contraceptive utilization in rural Ethiopia. *PLOS ONE*, 12(11), p.e0187311.

15. Okwaraji YB, Webb EL, Edmond KM. Barriers in physical access to maternal health services in rural Ethiopia. BMC Health Serv Res. 2015; 15:493.

16. Aparna Sundaram RH, Zeba Sathar, Sabahat Hussain, Weissman EPaE. Adding It Up: Costs and Benefits of Meeting the Contraceptive and Maternal and Newborn Health Needs of Women in Pakistan. Population Council. 2019

17. Sumera Aziz Ali SS, Neelofar Sami, Mir Shabbar, Muhammad Ahmed. Geographic Access to Working Family Planning Centers and Unintended Pregnancies among Married Women: A Community Based Nested Case Control Study. Open Journal of Epidemiology. 2016.

18. Sumera Aziz Ali ZF. Predictors of modern contraceptive usage among women in rural Pakistan physical access to services still a major barrier. EC Gynecology. 2017.

19. Digitale J, Psaki S, Soler-Hampejsek E, Mensch BS. Correlates of Contraceptive Use and Health Facility Choice among Young Women in Malawi. Ann Am Acad Pol Soc Sci. 2017;669(1):93-124.

20. Aviisah PA, Dery S, Atsu BK, Yawson A, Alotaibi RM, Rezk HR, et al. Modern contraceptive use among women of reproductive age in Ghana: analysis of the 2003-2014 Ghana Demographic and Health Surveys. BMC Womens Health. 2018;18(1):141.

21. Rios-Zertuche D, Blanco LC, Zúñiga-Brenes P, Palmisano EB, Colombara DV, Mokdad AH, et al. Contraceptive knowledge and use among women living in the poorest areas of five Mesoamerican countries. Contraception. 2017;95(6):549-57.

22. Basu, A. M. (2005). Ultramodern contraception. *Asian Population Studies*, *1*(3), 303–323. doi:10.1080/17441730500441178

23. Hossain S, Sripad P, Zieman B, Roy S, Kennedy S, Hossain I, et al. Measuring quality of care at the community level using the contraceptive method information

For peer review only - http://bmjopen.bmj.com/site/about/guidelines.xhtml

index plus and client reported experience metrics in Bangladesh. J Glob Health. 2021; 11:07007.

24. Culwell K, Hillard, P, Glob. Patient education and contraceptive compliance. 2008

25. Nisar S, Khan M, Nisar U. Impact Of Lady Health Workers On The Contraceptive Prevalence Rate In District Mardan. J Ayub Med Coll Abbottabad. 2020;32(1):104-10.

26. Tibaijuka L, Odongo R, Welikhe E, Mukisa W, Kugonza L, Busingye I, et al. Factors influencing use of long-acting versus short-acting contraceptive methods among reproductive-age women in a resource-limited setting. BMC Womens Health. 2017;17(1):25.

27. Westhoff CL, Heartwell S, Edwards S, Zieman M, Stuart G, Cwiak C, et al. Oral contraceptive discontinuation: do side effects matter? Am J Obstet Gynecol. 2007;196(4): 412.e1-6; discussion. e6-7.

For peer review only

Figure1: Map of Gilgit Baltistan showing distance in Kilometers from union council to family planning centers and distribution of LHWs in public and private family planning centers.

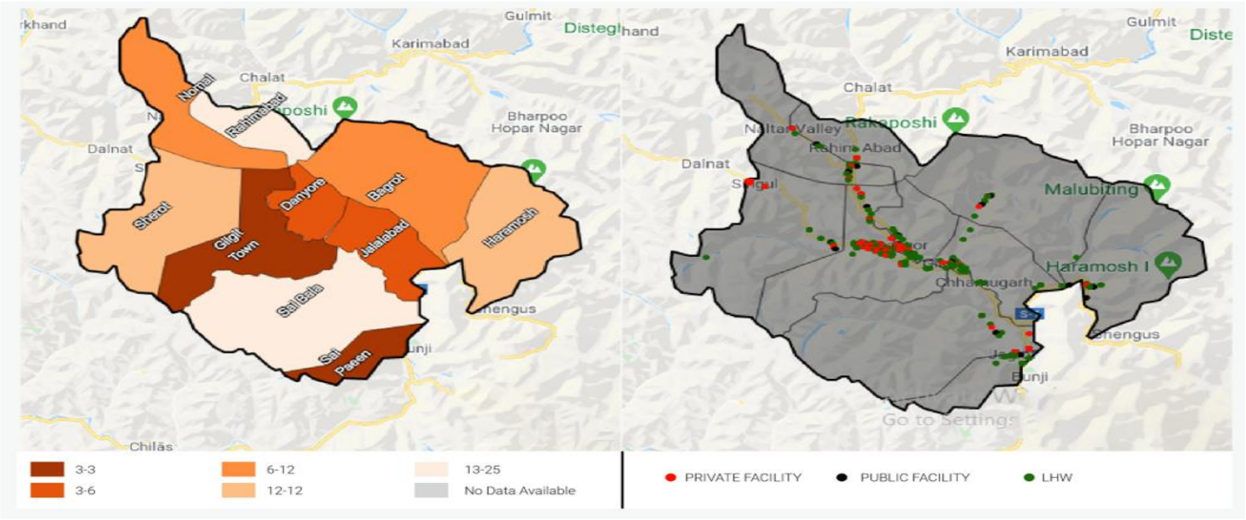

peer review only

STROBE Statement—Checklist of items that should be included in reports of ***case-control studies***

	Item No	Recommendation	Page No
**Title and abstract**	1	Indicate the study's design with a commonly used term in the title or the abstract<br>Case Control Study	
		(*b*) Provide in the abstract an informative and balanced summary of what was done and what was found<br>This study aimed to determine the factors associated with continuation of hormonal contraceptive methods among married women of Gilgit, Pakistan at least six months after their initiation. This was an Unmatched case control study conducted in the Community settings of district Gilgit, Pakistan from April 1, 2021, to July 30, 2021.The cases were married women of reproductive age who at the time of interview were using a hormonal method of contraception for at least last six months continuously, and controls were married women of reproductive age who had used hormonal method in the past and currently were using a non-hormonal method for at least last six months. Primary and Secondary outcome measures was OR for continuation of hormonal contraceptive.<br><br>The factors significantly associated with continuous use of hormonal contraceptive methods for our sample from Gilgit were family planning center at the walking distance from home (AOR 6.33, 95% CI, 3.74-10.71), satisfaction with current method in use (AOR 3.64 95% CI, 2.06-6.44), visits to the family planning center to avail services (AOR 1.86 95% CI, 1.07-3.45) and relatively older age of women (AOR 1.07, 95% C1, 1.02-1.12). In addition, women with formal education (AOR 0.27 95% CI, 0.12-0.6), were less likely to use a modern contraceptive method.<br><br>Continuation of a hormonal method use was associated with easy access to family planning centers, satisfaction with the current method use and frequent visits to the family planning centers. Continuation of hormonal method use was also seen in women with low education status. The importance of the presence of family planning centers near residential areas cannot be emphasized more. This not only provides easy access to FP methods, but also reassures women for continuation of modern methods when they face any unpleasant effects while using these.	
**Introduction**			
Background/rationale	2	Explain the scientific background and rationale for the investigation being reported.<br>Factors associated with contraceptive continuation are both methods related such as efficacy of the method, frequency of doses, and client- related such as age, education level, parity etc. Distance of the family planning center from home and availability of different methods at the center are additional important factors contributing to continuity in use of a method. The presence of healthcare staff at the center, level of education, age, number of living children and area of residence were all found to be associated with contraceptive continuation. Information regarding predictors of continuation of hormonal contraceptives is scarce from Pakistan and information related to continuation of any contraceptive method is mostly unavailable in Pakistan. Findings from this study would help in improving contraceptive prevalence rate and decreasing discontinuation of hormonal methods which are more effective compared to non-hormonal method. Moreover, promoting factors that are associated with the continuation of hormonal method will help to avoid	

For peer review only - http://bmjopen.bmj.com/site/about/guidelines.xhtml

		untimed and unwanted pregnancies hence it will improve maternal health.	
Objectives	3	State specific objectives, including any prespecified hypotheses.<br>This study aimed to determine the factors associated with continuation of hormonal contraceptive methods among married women of Gilgit, Pakistan at least six months after their initiation.<br>**Hypothesis**<br>The women who use hormonal methods continuously for six months are two times more at Odds of living at less than 30 minutes walking distance from a FP center as compared to those who discontinue to use a method within six months of starting it.	
**Methods**			
Study design	4	Present key elements of study design early in the paper<br>We use a community-based unmatched case control study design, with ratio of cases to control as 1:2.	
Setting	5	Describe the setting, locations, and relevant dates, including periods of recruitment, exposure, follow-up, and data collection.<br>We collected data from the catchment population of LHWs of 3 union councils Gilgit town, Danyore, and Nomal. Data were collected from March 2021 to July 2021 by lady health visitors who were trained for data collection.	
Participants	6	Give the eligibility criteria, and the sources and methods of case ascertainment and control selection. Give the rationale for the choice of cases and controls<br>Hormonal method users were sexually active women who were using one of the hormonal contraceptive methods i.e., pills, injectables or implants for at least last six months without disruption at the time of enrolment in the study and Controls were sexually active married women of reproductive age, with history of using a hormonal method but stopped it before six months after initiating it and switched to a non-hormonal contraceptive method for at least last six months. We excluded women using intrauterine contraceptive device (IUCD), as there are of two types (hormonal and non-hormonal) methods and women might not know the type of IUCD she was using. Women who were using permanent method of contraception were also excluded	
		(*b*) For matched studies, give matching criteria and the number of controls per case	
Variables	7	Clearly define all outcomes, exposures, predictors, potential confounders, and effect modifiers. Give diagnostic criteria, if applicable<br>Contraceptive continuation is the process of uninterrupted usage of contraceptive methods for at least six months.	
Data sources/ measurement	8*	For each variable of interest, give sources of data and details of methods of assessment (measurement). Describe comparability of assessment methods if there is more than one group<br>The study Questionnaire was designed after reviewing literature, PDHS 2017-18 questionnaire and service provision assessment survey form of Measure. The Questionnaire was translated into Urdu to make it understandable for study participants. We used Urdu as our medium of collecting information because it is our national language which is understood and spoken by a large majority (66.9 %) of young people in in GB (MICS 2017).	

For peer review only - http://bmjopen.bmj.com/site/about/guidelines.xhtml

Bias	9	Describe any efforts to address potential sources of bias	
Study size	10	Explain how the study size was arrived at<br>**Sample size estimation**<br>We used open epi software to estimate sample size of the study. The prevalence of various factors [(distance (32.12%), counselling provided by healthcare providers (62.77%), and experienced side effects (80.29)] for controls and cases were obtained from the literature search. We calculated sample size for all three factors mentioned and have selected range that is covering all three factors. To account for non-response rate we have increased sample size by 20% so, to determine an odds ratio of at least 2, by taking power of 80 percent and at significance of 5 percent with the ratio of 1:2 between cases and controls, a minimum of 141 cases and 282 controls were required for this study using 32.12 percent exposure in controls from literature.	
Quantitative variables	11	Explain how quantitative variables were handled in the analyses. If applicable, describe which groupings were chosen and why<br>Descriptive statistics were calculated for both categorical and continuous variables. Frequency and percentages have been reported for qualitative variables such as area of residence, level of education, socioeconomic status, cost of services, mode of transport, distance from home to FP center, decision maker of contraceptive use, source of current method, information given by LHW, satisfaction with home services, FP counselling and information related to side effects experienced by women. Mean and standard deviation is reported for quantitative variables such as age of woman, age at first contraceptive use, number of living children and number of FP center operational days.	
Statistical methods	12	Describe all statistical methods, including those used to control for confounding<br>Binary logistic regression	
		(*b*) Describe any methods used to examine subgroups and interactions	
		(*c*) Explain how missing data were addressed<br>there were no significant missing values	
		(*d*) If applicable, explain how matching of cases and controls was addressed	
		(*e*) Describe any sensitivity analyses	
**Results**			
Participants	13*	Report numbers of individuals at each stage of study—eg numbers potentially eligible, examined for eligibility, confirmed eligible, included in the study, completing follow-up, and analysed<br>number of potential eligible: 520<br>examined for eligibility: 499<br>confirm eligible:423	
		(b) Give reasons for non-participation at each stage	
		(c) Consider use of a flow diagram	
Descriptive data	14*	Give characteristics of study participants (eg demographic, clinical, social) and information on exposures and potential confounders<br>For cases, we enrolled 141 women who were using one of the hormonal	

For peer review only

For peer review only - http://bmjopen.bmj.com/site/about/guidelines.xhtml

		methods (Pills, injections and Implants) for last six months without disruption and 282 women who ever used hormonal method in past and currently were using non-hormonal method from last six months (discontinued users). Among cases, 24.1 percent of the women were using pills, 2.1 percent of the women were using implant and 73.4 percent were using injectables for contraception. Traditional method was more common among controls. 59.2 percent of the women were using traditional methods and 40.7 percent of the women were using condoms. Previously, 62 percent of the women among controls discontinued pills, 0.35 percent discontinued implant and 37.5 percent of the women discontinued injectables and started to use non-hormonal method.	
		(b) Indicate number of participants with missing data for each variable of interest	
Outcome data	15*	Report numbers in each exposure category, or summary measures of exposure	

For peer review only

For peer review only - http://bmjopen.bmj.com/site/about/guidelines.xhtml

Main results	16	(*a*) Give unadjusted estimates and, if applicable, confounder-adjusted estimates and their precision (eg, 95% confidence interval). Make clear which confounders were adjusted for and why they were included	
		(*b*) Report category boundaries when continuous variables were categorized	
		(*c*) If relevant, consider translating estimates of relative risk into absolute risk for a meaningful time period	
Other analyses	17	Report other analyses done—eg analyses of subgroups and interactions, and sensitivity analyses	
**Discussion**			
Key results	18	Summarise key results with reference to study objectives<br>the model we concluded that odds of a walking distance of at most 30 minutes from home to a family planning center is 6.33 times more among continued users compared to discontinued users (95% CI=3.74-10.71). For every one-year increase in age of the women among continued users, the increase in log odds of hormonal contraceptive continuation was 1.07 times compared to discontinued users (adjusted OR=1.07, CI=1.02-1.12). Odds of having formal education was 73% less among continued users compared to discontinued users (adjusted OR=0.27, 95% CI=0.07-0.89). Odds of being satisfied with current method was 3.64 times more among continued users compared to discontinued users (95% CI=2.06-6.44). Odds of visiting to family planning center was 1.86 times more among continued users compared to discontinued users (95% CI=1.07-3.45).	
Limitations	19	Discuss limitations of the study, taking into account sources of potential bias or imprecision. Discuss both direction and magnitude of any potential bias<br>Major limitation of the study was Subjective assessment of Primary exposure (taken on face value) due to unavailability of internet access in all union councils of district Gilgit. Women might have given wrong responses. Furthermore, this study is generalizable to areas that are geographically similar to district Gilgit.	
Interpretation	20	Give a cautious overall interpretation of results considering objectives, limitations, multiplicity of analyses, results from similar studies, and other relevant evidence<br>In Gilgit, continuation of hormonal method use was associated with easy access to family planning centers, satisfaction with current method use and visits to FP center. In addition, continuation was also seen in women with no formal education. Women who were using hormonal method continuously were three years older than discontinued users. Despite experiencing side-effects women who were using hormonal contraceptives continued to use method of their choice for at least six months.	
Generalisability	21	Discuss the generalisability (external validity) of the study results<br>this study is generalizable to areas that are geographically similar to district Gilgit.	
**Other information**			
Funding	22	Give the source of funding and the role of the funders for the present study and, if applicable, for the original study on which the present article is based<br>Funding from HRP Alliance, part of UNDP-UNFPA-UNICEF-WHO-World Bank special program of research, development, and Research Training in Human Reproduction (HRP), a cosponsored program executed by The World Health Organization (WHO). The main funders HRP had no role in study design, data collection and analysis.	

*Give information separately for cases and controls.

For peer review only - http://bmjopen.bmj.com/site/about/guidelines.xhtml

Note: An Explanation and Elaboration article discusses each checklist item and gives methodological background and published examples of transparent reporting. The STROBE checklist is best used in conjunction with this article (freely available on the Web sites of PLoS Medicine at http://www.plosmedicine.org/, Annals of Internal Medicine at http://www.annals.org/, and Epidemiology at http://www.epidem.com/). Information on the STROBE Initiative is available at http://www.strobe-statement.org.

For peer review only

For peer review only - http://bmjopen.bmj.com/site/about/guidelines.xhtml
